# Interleukin-13 and its receptor are synaptic proteins involved in plasticity and neuroprotection

**DOI:** 10.1038/s41467-023-35806-8

**Published:** 2023-01-13

**Authors:** Shun Li, Florian olde Heuvel, Rida Rehman, Oumayma Aousji, Albrecht Froehlich, Zhenghui Li, Rebecca Jark, Wanhong Zhang, Alison Conquest, Sarah Woelfle, Michael Schoen, Caitlin C. O´Meara, Richard Lee Reinhardt, David Voehringer, Jan Kassubek, Albert Ludolph, Markus Huber-Lang, Bernd Knöll, Maria Cristina Morganti-Kossmann, Marisa M. Brockmann, Tobias Boeckers, Francesco Roselli

**Affiliations:** 1 Department of Neurology, Ulm University, Ulm, Germany; 2 Department of Neurosurgery, Kaifeng central Hospital, Kaifeng, China; 3 Centre for Molecular Neurobiology Hamburg, University Medical Centre Hamburg-Eppendorf, Hamburg, Germany; 4 National Trauma Research Institute and Department of Neurosurgery, TheAlfred Hospital, Melbourne, VIC Australia; 5 Institute of Anatomy and Cell Biology, Ulm University, Ulm, Germany; 6 Cardiovascular Centre, Medical College of Wisconsin, Milwaukee, WI USA; 7 Department of Immunology and Microbiology, University of Colorado Medical School, Aurora, CO USA; 8 Department of Infection Biology, Friedrich-Alexander University Erlangen-Nuremberg (FAU), Erlangen, Germany; 9 German Centre for Neurodegenerative Diseases (DZNE)-Ulm, Ulm, Germany; 10 Institute of Clinical and Experimental Trauma Immunology, Ulm University, Ulm, Germany; 11 Institute of Neurobiochemistry, Ulm University, Ulm, Germany; 12 Department of Epidemiology and Preventive Medicine, Monash University, Melbourne, VIC Australia

**Keywords:** Neuroscience, Diseases of the nervous system

## Abstract

Immune system molecules are expressed by neurons, yet their functions are often unknown. We have identified IL-13 and its receptor IL-13Ra1 as neuronal, synaptic proteins in mouse, rat, and human brains, whose engagement upregulates the phosphorylation of NMDAR and AMPAR subunits and, in turn, increases synaptic activity and CREB-mediated transcription. We demonstrate that increased IL-13 is a hallmark of traumatic brain injury (TBI) in male mice as well as in two distinct cohorts of human patients. We also provide evidence that IL-13 upregulation protects neurons from excitotoxic death. We show IL-13 upregulation occurring in several cohorts of human brain samples and in cerebrospinal fluid (CSF). Thus, IL-13 is a physiological modulator of synaptic physiology of neuronal origin, with implications for the establishment of synaptic plasticity and the survival of neurons under injury conditions. Furthermore, we suggest that the neuroprotection afforded through the upregulation of IL-13 represents an entry point for interventions in the pathophysiology of TBI.

## Abstract (web summary)

Il-13 is expressed in neurons and IL-13 ko causes memory impairment. Here, authors show that IL-13 and its receptor IL-13Ra1 are pre- and post-synaptic proteins, respectively, involved in synaptic signaling, plasticity and neuroprotection.

## Introduction

Continuous remodeling of synapses at the structural and functional level is critical not only to the formation and retention of new memories and to the learning process, but also to the acute and long-term response to pathological conditions. Several components of the innate^1–3^ and adaptive^4^ immune systems have surprisingly been shown to be involved in synaptic plasticity^5^, either independent of their primary immune function^2,4,6^ or in relation to the modulation of local microglia^1^. These immune-related mediators originate from glial cells^2,6^ and neurons themselves^7,8^. Nevertheless, synapses may also respond to the large amounts of mediators secreted by infiltrating inflammatory cells during inflammation^9^ or in TBI^10^.

Recent evidence^8,11^ suggests that IL-13 is expressed in neurons in the healthy brain and is upregulated upon injury. In normal conditions IL-13 may be involved in neuromodulation and in the synaptic plasticity underlying spatial memory and learning^12^. In the immune system, IL-13 is secreted by mast cells, basophils and eosinophils, as well as CD4 T cells, ILC2 and NK T cells^13^, orchestrating the response of subsets of Th2 lymphocytes and driving the IgE switch in B lymphocytes^14^. IL-13 signals through a type-I receptor, comprising of the specific IL-13Ra1 subunit that, upon ligand binding, forms a heterodimer with IL-4 receptor alpha chain and drives a JAK2/Tyk2-dependent phosphorylation of the transcription factor STAT6^15^. The high-affinity type-II receptor IL-13Ra2 does not have a cytoplasmic chain but does contribute to signaling cascades^16^.

IL-13 acts on epithelial cells and smooth muscle cells and it is involved as an effector cytokine in the pathogenesis of allergic diseases such as asthma^17^ and atopic dermatitis^18,19^. Blockade of IL-13 by monoclonal antibodies is a therapeutic option in these conditions^20,21^.

In this study, we have investigated the previously unexplored biology of neuronal IL-13. We demonstrate that IL-13 and IL-13Ra1 are synaptic components expressed in rat, mouse, and human neurons in an activity-dependent manner in normal conditions and, most notably, that neuronal IL-13 is upregulated upon TBI. Furthermore, we show that IL-13 triggers the phosphorylation of glutamate receptors and several presynaptic proteins; it increases synaptic activity and neuronal firing ultimately driving the phosphorylation of several transcription factors, including CREB. Finally, we reveal that IL-13 protects neurons against excitotoxic insults, implying a direct neuroprotective role in TBI. We have used human samples of brain and CSF from three distinct normal and TBI cohorts to demonstrate the relevance of IL-13 in the physiopathology of TBI in patients.

## Results

### IL-13 and its receptor IL-13Ra1 are neuronal synaptic proteins

**Fig. 1:**
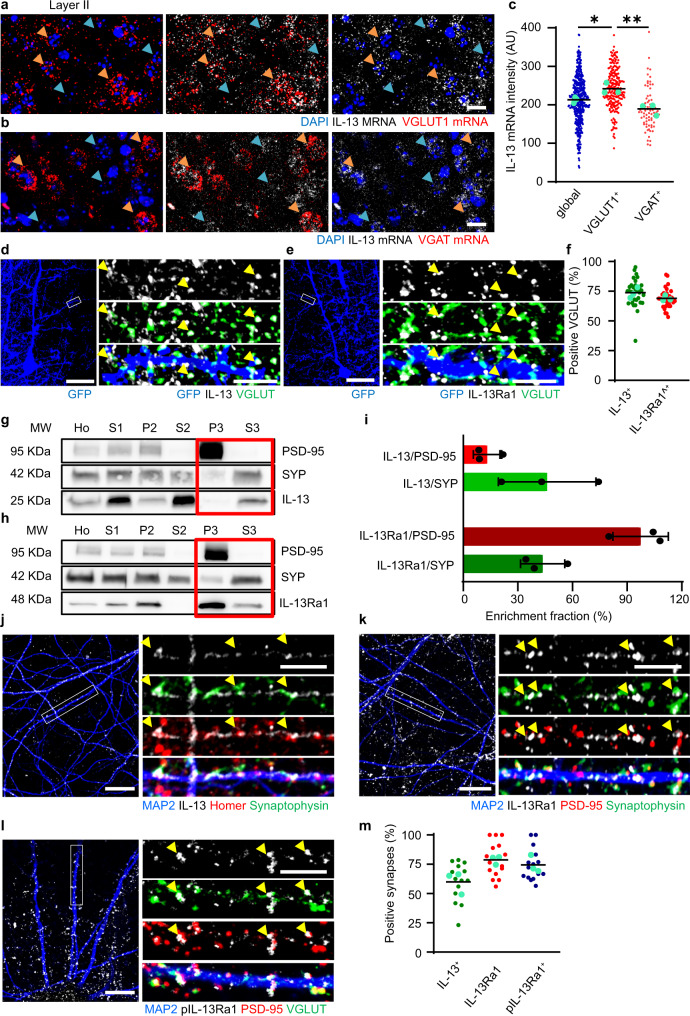
Neuronal IL-13 and its receptor IL-13Ra1 are synaptic proteins. **a**–**c** Increased *IL-13* mRNA intensity in VGLUT1 positive population (orange arrows, **a**) compared to VGAT positive (orange arrows, **b**; *p* = 0.0049) and global cell populations (blue arrows; *p* = 0.0481) in layer II/III of mouse cortical sections (single molecule in situ hybridization). *N* = 3; *n* = global: 425; VGLUT1+: 214; VGLUT2+: 72 neurons. Scale bar: 20 μm. *: *p* < 0.05; **: *p* < 0.01. AU: arbitrary units. **d**–**f** Synaptic localization of IL-13 in mouse cortical sections (GFP sparse labelling and Immunostaining with GFP, pan-VGLUT and IL-13/IL-13Ra1). IL-13 and IL-13Ra1 show 74% and 69% respectively, of colocalization with VGLUT positive synapses. *N* = 3; *n* = IL-13: 31; IL-13Ra1: 30 dendrites. Scale bar overview 50 μm and insert 5 μm. **g**–**i** Fractionation experiment in mouse cortical tissue shows IL-13 and IL-13Ra1 localization in homogenates (Ho), homogenates without nuclei, cell debris and extracellular matrix (S1), crude membrane fraction (P2), cytosolic compartment (S2), postsynaptic density (P3) and presynaptic cytosol fraction (S3). Enriched fractions show a lower amount of IL-13 in the PSD-95 positive fraction (13%) compared to the synaptophysin positive fraction (46%). Enriched fractions show a higher amount of IL-13Ra1 in the PSD-95 positive fraction (97%) compared to the synaptophysin positive fraction (44%). *N* = 3. Data shown as mean ± SD. **j**–**m** Synaptic localization of IL-13, IL-13Ra1 and pIL-13Ra1 in rat cortical neurons (Immunostaining with MAP2, pre- and postsynaptic markers). IL-13, IL-13Ra1 and pIL-13Ra1 show 60%, 79% and 75%, respectively, of colocalization with mature synapses. *N* = 3; *n* = IL-13 + : 14; IL-13Ra1 + : 15; pIL-13Ra1: 15 dendrites. Scale bar overview 10 μm and insert 5 μm. **c** One-way ANOVA with Sidak’s multiple comparison. Source data are provided as a Source Data file.

Recent reports hinted at substantial expression of IL-13 in the brain^8,11^. We set out to establish the source(s) and the localization of IL-13 and its receptor in the cerebral cortex of the mouse. By in situ hybridization we surprisingly found that a substantial fraction of cells expressed abundant levels of *IL-13* mRNA; double in situ hybridization using markers of glutamatergic neurons (*VGLUT1* for layer II/III, *VGLUT2* for layer IV;^22^, Fig. 1a and Supplementary Fig. 1a, b) and GABAergic neurons (*VGAT*, Fig. 1b) revealed that *IL-13* is expressed in both cell subpopulations. Compared to the overall cortical population (blue arrows) VGLUT1 + glutamatergic neurons (orange arrows, panel a) expressed higher levels of *IL-13* than GABAergic neurons (orange arrows, panel b) implying that *IL-13* was more strongly expressed in excitatory neurons (Fig. 1c). Interestingly, *IL-13* was also expressed in VGLUT1+ and in VGLUT2 + neurons in Layer IV (orange arrows; Supplementary Fig. 1a–c). Notably, a substantial degree of variability in expression levels was observed across glutamatergic neurons (Fig. 1c and Supplementary Fig. 1a–c). Secondly, we investigated the localization of IL-13 protein and its cognate receptor IL-13Ra1 in vivo by immunostaining sparse GFP-labelled neurons. AAV2 expressing cytoplasmic GFP (to outline the shape of the cell) was injected in mouse somatosensory cortex, later immunostained for the presynaptic marker pan-VGLUT and either IL-13 or IL-13Ra1. Approximately 70% of bona fide VGLUT + synapses showed immunoreactivity for IL-13 (74 ± 11%, Fig. 1d, f and Supplementary Fig. 1f) or, in independent experiments, for IL-13Ra1 (69 ± 8%, Fig. 1e, f and Supplementary Fig. 1g). We then sought an additional confirmation of the synaptic nature of IL-13 and IL-13Ra1 using a brain fractionation protocol to isolate distinct cellular subcompartments (neuronal membranes, synaptosomes, postsynaptic membranes and presynaptic vesicles^23,24^). The enrichment of the individual fractions was monitored by detecting PSD-95 and Synaptophysin as markers of the postsynaptic and the vesicle fractions respectively, by Western Blot. IL-13 immunoreactivity was strongly enriched in the synaptic vesicle fraction (S3) and mostly excluded from the postsynaptic fraction (P3), similar to synaptophysin (Fig. 1g; enrichment fraction IL-13/SYP and IL-13/PSD-95 shown in Fig. 1i). Interestingly, IL-13Ra1 displayed a very strong enrichment in the postsynaptic fraction (mirroring PSD-95, Fig. 1h; enrichment fraction IL-13Ra1/SYP and IL-13Ra1/PSD-95 shown in Fig. 1i) although a smaller amount could be detected in the S3 fraction. Thus, these findings not only confirm the synaptic localization of IL-13 and IL-13Ra1 but suggest a pre- and postsynaptic (respectively) enrichment.

Thirdly, since the bulk cortical tissue has a very complex architecture with a high density of intertwined astrocytes and neuronal processes, we set out to elucidate the synaptic localization of both IL-13 and IL-13Ra1 in cultured rat cortical neurons. In E18-DIV21 cultures (which contain ~6% astrocytes and no microglia) we detected a robust expression of mRNA for *IL-13*, *IL-13Ra1* and *IL-13Ra2* (Supplementary Fig. 1e). Cultured neurons displayed a large number of synaptic contacts (PSD-95+/Homer+ and pan-VGLUT+/Synaptophysin+) along MAP2 + dendrites with ~70% of PSD-95+/VGLUT+ or Homer+/Synaptophysin+ synapses immunoreactive for IL-13, IL-13Ra1 or phosphorylated IL-13Ra1 (Fig. 1j–m). To control for the specificity of the anti-IL-13 mouse monoclonal antibody, we repeated this set of experiments with an unrelated rabbit polyclonal antibody against IL-13, obtaining a closely matched pattern (Supplementary Fig. 1d). We further validated the mouse monoclonal and rabbit polyclonal anti-IL-13 antibodies using brain tissue from *IL-13*^*−/*−^ mice^25^: both antibodies generated negligible staining in the *IL-13*^*−/*−^ samples (Supplementary Fig. 1h, i).

### Super-resolution microscopy reveals pre- and postsynaptic localization of IL-13 and IL-13Ra1

**Fig. 2:**
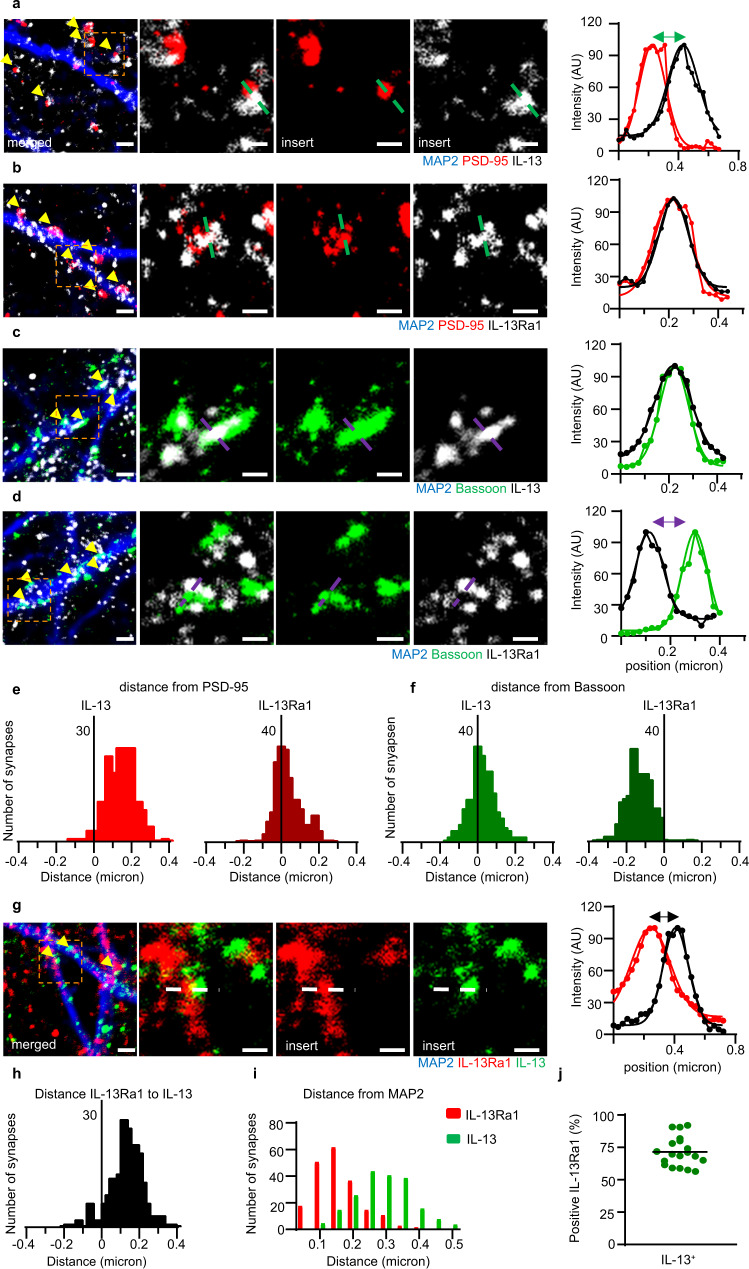
Presynaptic IL-13 and a postsynaptic lL-13Ra1 demonstrated by super-resolution microscopy. **a** Distinct separation between PSD-95 and IL-13 visible in STED images of single synapses. **b** STED imaging and intensity profile plots of single synapses show an overlap between PSD-95 and IL-13Ra1. **c** STED imaging and intensity profile plots of single synapses show an overlap between Bassoon and IL-13. **d** Distinct separation between Bassoon and IL-13Ra1 visible in STED images of single synapses. **e** Distribution of distance between IL-13 and IL-13Ra1 peaks compared to PSD-95 reveals that IL-13 peak is distal to PSD-95, the IL-13Ra1 overlaps with the PSD-95 peak. **f** Distribution of distance between peaks reveal an overlap between IL-13 and Bassoon, whereas IL-13Ra1 peak is distinct at −150 nm from Bassoon. **g** STED imaging and intensity profile of single synapses show a separation between IL-13Ra1 and IL-13. **h** Distribution of distance of IL-13 and IL-13Ra1 reveals that the two peaks do not overlap and are located at ~150 nm. **i** Distribution of the separation of IL-13Ra1 and IL-13 in reference to the MAP2 + dendritic profile reveals that IL-13Ra1 peak is proximal, whereas IL-13 peak is distally located, in agreement with post- and presynaptic localization. **j** IL-13 shows 72% synaptic colocalization with IL-13Ra1. **a**–**j** In all experiments *N* = 3; *n* = 200 synapses (19 dendrites for **j**). Scale bar overview: 1 μm, scale bar insert: 500 nm. AU: arbitrary units. Source data are provided as a Source Data file.

Since the resolution of the diffraction-limited confocal microscopy prevented the establishment of pre- or postsynaptic nature for IL-13 and its receptor, we opted to use a STED nanoscopic imaging paradigm to discern their localization. We preliminarily validated our ability to distinguish closely apposed synaptic structures by successfully imaging the separation of two sets of pre- and postsynaptic markers (Bassoon and Homer; Synaptophysin and PSD-95) in cultured cortical neurons (Supplementary Fig. 2a–d). Furthermore, we showed that Bassoon puncta immunostained with a single mouse anti-Bassoon primary antibody and two anti-mouse secondary antibodies conjugated to two distinct fluorochromes, produced STED images with almost complete peak overlay, indicating no systematic shift in the imaging setup. Next, we immunostained cultured cortical neurons for PSD-95 or Bassoon together with IL-13 or IL-13Ra1. We plotted the relative distribution of the peaks of IL-13 relative to either PSD-95 or Bassoon (using the MAP2 profiles to establish the synaptic polarity^26,27^). Most notably, the peak of IL-13 distribution was located approximately 150 nm away from the PSD-95 peak (*n* = 200 synapses; Fig. 2a and e). On the contrary, IL-13 peak overlapped with the Bassoon peak (*n* = 200 synapses; Fig. 2c and f). These findings corroborated the presynaptic nature of IL-13. Conversely, the peak of IL-13Ra1 immunoreactivity largely overlapped with the PSD-95 peak on the postsynaptic side (*n* = 200 synapses; Fig. 2b and e); a close inspection of the STED images revealed that in some cases multiple IL-13Ra1 clusters were localized within the PSD-95+ cluster (Supplementary Fig. 2e). In contrast, the IL-13Ra1 peak was localized ~150 nm away (toward the dendrite) from the Bassoon peak (Fig. 2d and f). In agreement with the colocalization measures obtained in confocal images, IL-13 and IL-13Ra1 colocalized with Bassoon and PSD-95 in ~75% of synapses (Supplementary Fig. 2g). Finally, we directly contrasted IL-13 and IL-13Ra1 immunolocalization in STED microscopy. The two corresponding peaks did not overlap, but, relative to the MAP2 profile, were shifted ~150 nm (*n* = 200 synapses; Fig. 2g–i); 72% of IL-13 clusters colocalized with IL-13Ra1 clusters (Fig. 2j). Thus, the STED imaging data provide strong evidence for a presynaptic localization of IL-13 and a (mainly) postsynaptic localization of IL-13Ra1 in cultured neurons, in remarkable agreement with the fractionation data from the bulk brain. However, the existence of a smaller, presynaptic pool of IL-13Ra1 cannot be currently discounted.

Taken together, this data (Fig. 1 and Fig. 2) identifies IL-13 and IL-13Ra1 as neuronal, synaptic proteins in rat and mouse brains and point toward a polarized biology with IL-13 released by presynaptic terminals acting on postsynaptic IL-13Ra1 receptors.

### IL-13 triggers large-scale phosphorylation of glutamate receptors and presynaptic proteins

To gain insights into the synaptic functions of IL-13, we used phospho-antibody arrays to characterize the large-scale architecture of phosphorylation events in neurons set in motion by the cytokine. We treated cultured cortical neurons with IL-13 (or vehicle) for 1 h or 3 h; whole-cell protein extract was then processed using a glass-based phospho-antibody array assay targeting multiple phosphorylation sites on 167 distinct proteins (including ion channels, neurotransmitter receptors, vesicle proteins and cytoskeletal elements, among the others).

**Fig. 3:**
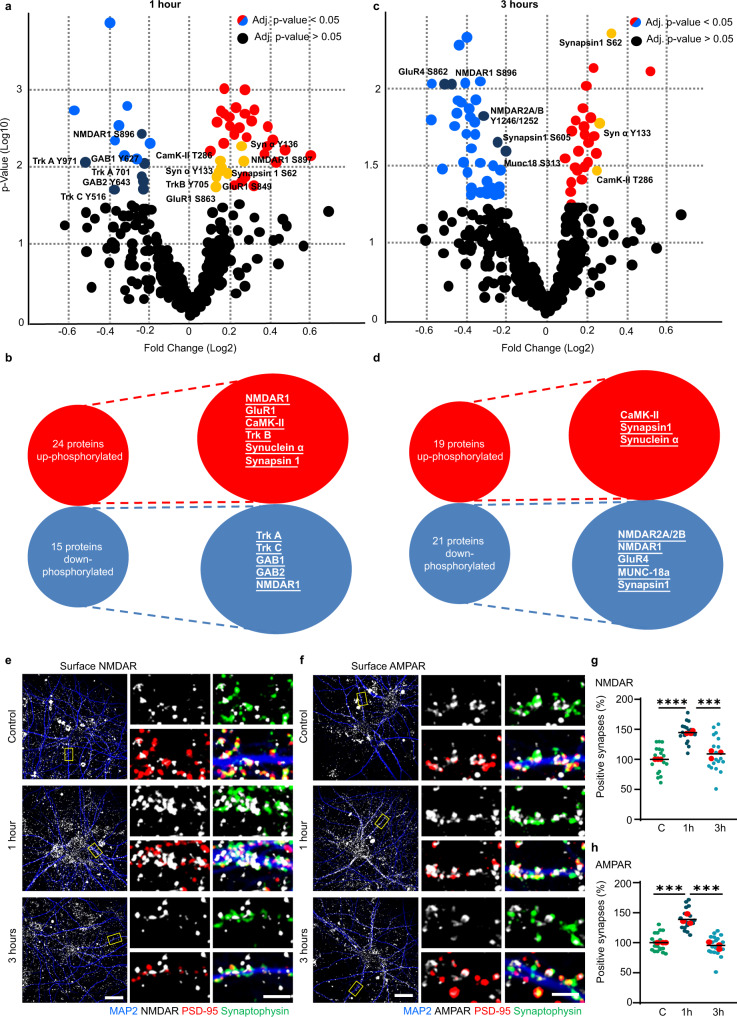
IL-13 causes the large-scale phosphorylation of glutamate receptors and presynaptic proteins. **a**, **b** Volcanoplot and list of proteins showing significant change in their phosphorylation (up or down) 1 h after neurons were exposed to IL-13 (50 ng/ml) or control (0.1% BSA). red = up-phosphorylated, blue = down-phosphorylated. *N* = 4. **c**, **d** Volcanoplot and list of proteins showing significant change in their phosphorylation (up or down) 3 h after neurons were exposed to IL-13 (50 ng/ml) or control (0.1% BSA). red = up-phosphorylated, blue = down-phosphorylated. *N* = 4. Complete list of significantly phosphorylated proteins can be found in supplementary information. **e**–**g** Extracellular NDMAR antibody feeding assay co-stained with MAP2, Synaptophysin and PSD-95 in rat primary cortical neurons 1 h and 3 h after IL-13 treatment (50 ng/ml) or vehicle (0.1% BSA). Significant increase in surface NMDAR is visible 1 h (*p* < 0.0001) after IL-13 application. *N* = 3. Scale bar overview: 20 μm, scale bar insert: 2 μm. ***: *p* < 0.001; ****: *p* < 0.0001. **f**–**h** Extracellular AMPAR antibody feeding assay co-stained with MAP2, Synaptophysin and PSD-95 in rat primary cortical neurons 1 h and 3 h after IL-13 treatment (50 ng/ml) vs vehicle (0.1% BSA). Significant increase in surface AMPAR is visible 1 h (*p* = 0.0005) after IL-13 application. *N* = 3. Scale bar overview: 20 μm, scale bar insert: 2 μm. ***: *p* < 0.001 (**a**, **c**): PROTein array Expression AnalysiS; https://github.com/Rida-Rehman/PROTEAS. (**f**, **g**): One-way ANOVA with Sidak’s multiple comparison. Source data are provided as a Source Data file.

At 1 h post stimulation, 24/167 proteins displayed an increase in phosphorylation, whereas 15/167 proteins were down-phosphorylated on several serine, threonine or tyrosine phospho epitopes. Intriguingly, both pre- and postsynaptic proteins were significantly represented in the hit list. Among the postsynaptic proteins, IL-13 increased the phosphorylation of NMDAR1 (S897), of AMPAR1 (GluR1, both S849 and S863), of CaMKII (T286 and T305) and, interestingly, of the BDNF receptor TrKB (Y515 and Y705). Among the presynaptic proteins, IL-13 induced the phosphorylation of α-synuclein (Y133 and Y136) and synapsin I (S62). A substantial number of signaling cascades were also activated, including CaMKI and CaMKIV (S177 and S196/200, respectively), GSK3β (Y216/279), PP2A (Y307), PKA (T197) and GRK2 (S685). Other notable IL-13 up-phosphorylated proteins included cytoskeletal proteins Tau (S231) and Merlin (S518) (Fig. 3a, b and Supplementary Fig. 3a). Among the down-regulated phospho-epitopes were TrkA (Y680, Y701 and Y791) and TrkC (Y516) and the GAB family members GAB1 and GAB2 (Y627 and Y643, respectively); also, the NMDAR1 epitope S896 showed a degree of downregulation (Fig. 3a, b and Supplementary Fig. 3a).

The phosphorylation landscape at 3 h post stimulation displayed some degree of similarity with the 1 h, but also substantial differences. Overall, 19 proteins (out of 167 included in the array) were up-phosphorylated and 21 were down-phosphorylated. Again, both pre- and postsynaptic proteins were represented among the up-phosphorylated. Among the postsynaptic proteins, only CaMKII (T286) was still significantly up-phosphorylated. Among the presynaptic proteins, Synapsin I (S62) and α-synuclein (Y125 and Y133) were up-phosphorylated. A number of additional signaling proteins were still substantially up-phosphorylated, such as CaMKI (T177), GAB1 (Y659), DAB1 (Y220) and GRK2 (S29). Moreover, the cytoskeletal protein Tau (S235, S356, S422), Merlin (S10) and GAP43 (S41) were still strongly up-phosphorylated (Fig. 3c, d and Supplementary Fig. 3b). However, glutamate receptors were prominently represented among the proteins with decreased phosphorylation: NMDAR2A/2B (Y1246/1252), NMDAR1 (S895), AMPAR GluR4 (S862). Two presynaptic proteins appeared among the down-phosphorylated proteins, namely MUNC-18a (S313) and Synapsin I (S605). Several down-phosphorylated proteins were involved in protein degradation or post-translational processing, such as Parkin 1 (S131), Ataxin 1 (S776) and Preselinin-1 (S357). Other notable proteins displaying reduced phosphorylation included TrkA (Y791) and Doublecortin (S297) (Fig. 3c, d and Supplementary Fig. 3b).

Since IL-13 appeared to increase the phosphorylation of AMPAR and NMDAR subunits on epitopes related to surface expression and synaptic insertion^28–30^, we set out to confirm the surface localization of NMDAR and AMPAR upon IL-13 treatment, using antibodies directed against extracellular epitopes of either NMDAR (GluN1) or AMPAR (GluA1); synaptophysin and PSD-95 were used for synapse identification. IL-13 caused a significant increase in surface synaptic NMDAR after 1 h, although this effect disappeared 3 h after treatment (Fig. 3e, g). Likewise, surface synaptic AMPAR increased1 h after IL-13, with a subdued effect 3 h later (Fig. 3f, h).

Thus, IL-13 induced the rapid (1 h) phosphorylation of NMDA and AMPA-type glutamate receptors (and the corresponding surface trafficking of the receptors) and signaling proteins associated with synaptic plasticity (such as CaMKII and PKA), together with several presynaptic and cytoskeletal proteins. However, the phosphorylation of glutamate receptors was short-lived and at 3 h most glutamate receptors were down-phosphorylated (corresponding to removal from synaptic sites).

### IL-13 drives the phosphorylation of CREB and the induction of activity-dependent immediate-early genes

The pattern of increased phosphorylation of glutamate receptors and CamKII suggests that IL-13 may activate transcriptional programs associated with neuronal activity.

**Fig. 4:**
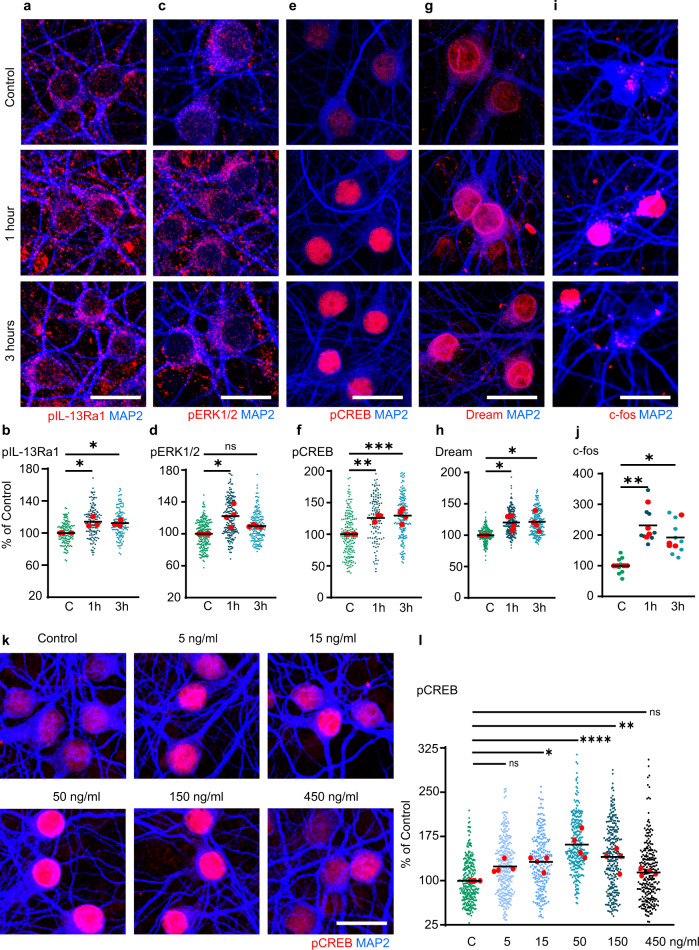
IL-13 induces CREB phosphorylation and immediate-early genes transcription. **a**, **b** Significant up-phosphorylation of IL-13Ra1 in rat cortical neurons 1 h (*p* = 0.0147) and 3 h (*p* = 0.0220) after IL-13 treatment (50 ng/ml). *N* = 3; *n* = C: 143; 1 h: 137; 3 h: 137 neurons. **c**, **d** Significant up-phosphorylation of ERK1/2 in rat cortical neurons 1 h (*p* = 0.0029) after IL-13 treatment (50 ng/ml). *N* = 4; *n* = C: 208; 1 h: 195; 3 h: 161 neurons. **e**, **f** Significant up-phosphorylation of CREB in rat cortical neurons 1 h (*p* = 0.0028) and 3 h (*p* = 0.0008) after IL-13 treatment (50 ng/ml). *N* = 3; *n* = C: 208; 1 h: 121; 3 h: 187 neurons. **g**, **h** Significant up-phosphorylation of DREAM in rat cortical neurons 1 h (*p* = 0.0381) and 3 h (*p* = 0.0387) after IL-13 treatment (50 ng/ml). *N* = 4; *n* = C: 232; 1 h: 243; 3 h: 246 neurons. **i**, **j** Significant increase of c-fos positive cells in rat cortical neurons 1 h (*p* = 0.0027) and 3 h (*p* = 0.0207) after IL-13 treatment (50 ng/ml). *N* = 4. **k**, **l** Dose-dependent effect of IL-13 treatment on CREB phosphorylation until 50 ng/ml, doses exceeding this limit reduces phosphorylation of CREB (C vs 5 ng/ml: *p* = 0.1089; C vs 15 ng/ml: *p* = 0.0219; C vs 50 ng/ml: *p* < 0.0001; C vs 150 ng/ml: *p* = 0.0052; C vs 450 ng/ml: *p* = 0.4619). *N* = 4; *n* = C: 274; 5 ng/ml: 292; 15 ng/ml: 232; 50 ng/ml: 283; 150 ng/ml: 265; 450 ng/ml: 265 neurons. In all experiments, vehicle (0.1% BSA) was used as control. Scale bar (**a**–**k**): 20 μm. *: *p* < 0.05; **: *p* < 0.01; ****: *p* < 0.0001. **b**, **d**, **f**, **h**, **j**, **l** One-way ANOVA with Dunnet’s multiple comparison. Source data are provided as a Source Data file.

As a preliminary step, we verified the activation of IL-13-triggered signaling cascades in neurons using experiments independent of antibody arrays. We immunostained cultured cortical neurons treated with IL-13 for 1 h and 3 h for phosphorylated IL-13Ra1 (Tyr405) and for phospho-ERK1/2 (Thr202/Tyr204). Compared to baseline, IL-13 triggered a significant elevation in the phosphorylation of IL-13Ra1 at 1 h and, albeit reduced in magnitude at 3 h as well (Fig. 4a, b). Of note, upregulation of phosphorylated IL-13Ra1 was observed also at the synaptic level: IL-13 treatment substantially increased, both the number of synapses (VGLUT+/PSD-95+) positive for pIL-13Ra1 and the immunofluorescence intensity for pIL-13Ra1 (Supplementary Fig. 4a–c).

Likewise, IL-13 triggered a strong elevation in ERK phosphorylation at 1 h, which subsided by 3 h (Fig. 4c, d).

Next, we focused on the phosphorylation of CREB, under the hypothesis that the increased phosphorylation of glutamate receptors upon IL-13 treatment may lead to increased neuronal firing. Indeed, IL-13 treatment caused a massive rise in the levels of phosphorylated CREB (S133) after 1 h and 3 h (compared to baseline; Fig. 4e, f; immunostaining was confirmed by Western blot, Supplementary Fig. 5a, b). In addition, following IL-13 treatment at 1 h and 3 h, we observed elevated phosphorylation of both STAT6 and STAT3 (Supplementary Fig. 5c–f), which are known targets of IL-13/IL-13Ra1^31^.

Finally, the elevation in pCREB levels was matched by the induction of the transcription of immediate-early genes (IEGs) and other activity-dependent genes. IL-13 upregulated nuclear ATF-3 (Supplementary Fig. 5g, h) and DREAM, after 1 h and 3 h treatments (Fig. 4g, h) and increased the number of c-fos-positive neurons (Fig. 4i, j). IL-13 treatment also upregulated the mRNA of several IEGs *(c-fos, fos-B, egr-1, egr-2, gadd45a and gadd45b;* Supplementary fig. 4i).

Most notably, when we treated neurons with increasing concentrations of IL-13 we did not obtain a linear increase in pCREB levels, but rather an inverse-U distribution: pCREB levels increased for IL-13 doses of 5–50 ng/ml (24%, 32% to 61% of vehicle, respectively) but then declined with doses up to 450 ng/ml (40% of baseline for 150 ng/ml and 14% for 450 ng/ml; Fig. 4k, l).

### IL-13 activates synaptic signaling cascades converging on CREB

**Fig. 5:**
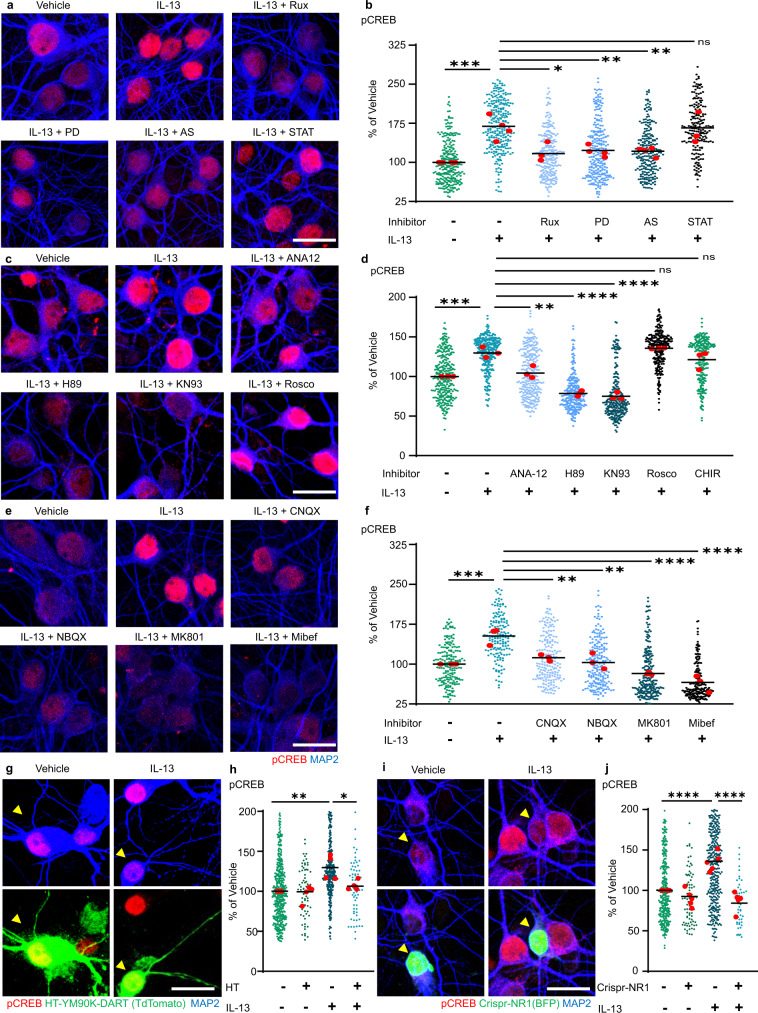
IL-13 activates CREB phosphorylation through JAK/ERK1/2 and NMDAR/AMPAR signaling. **a**, **b** JAK (Ruxolitinib; 280 nM), ERK1/2 (PD98059; 20 μM) and STAT6 (AS1517499; 1 μM) inhibitors significantly abolish the IL-13 dependent CREB phosphorylation in rat cortical neurons (veh vs IL-13: *p* = 0.0002; IL-13 vs RUX: *p* = 0.0108; IL-13 vs PD: *p* = 0.0083; IL-13 vs AS: *p* = 0.0082). STAT3 inhibitor (Stattic; 20 μM) did not alter the IL-13 induced phosphorylation of CREB (IL-13 vs STAT: *p* = 0.9994). *N* = 3–4; *n* = veh: 268; IL-13: 275; Rux: 249; PD: 249; AS: 248; STAT: 199 neurons. **c**, **d** TrkB receptor antagonist (ANA-12; 10 μM), PKA inhibitor (H89; 10 μM) and CAMK-II inhibitor (KN93; 10 μM) significantly reduce the IL-13 induced CREB phosphorylation (veh vs IL-13: *p* = 0.0002; IL-13 vs ANA-12: *p* = 0.0012; IL-13 vs H89: *p* < 0.0001; IL-13 vs KN93: *p* < 0.0001). Both the CDK5 inhibitor (Roscovitine; 10 μM) and GSK-3 inhibitor (CHIR 98014; 10 μM) do not alter IL-13 induced CREB phosphorylation (IL-13 vs Rosco: *p* = 0.8861; IL-13 vs CHIR: *p* = 0.5020). *N* = 3; *n* = Veh: 282; IL-13: 301; ANA-12: 276; H89: 253; KN93: 262; Rosco: 264; CHIR: 251 neurons. **e**, **f** AMPA receptor antagonists (CNQX, NBQX; both 10 μM), NMDA receptor antagonist (MK-801; 10 μM) and calcium channel blocker (Mibefradil; 10 μM) significantly reduce the IL-13 induced CREB phosphorylation (veh vs IL-13: *p* = 0.0004; IL-13 vs CNQX: *p* = 0.0037; IL-13 vs NBQX: *p* = 0.0010; IL-13 vs MK801: *p* < 0.0001; IL-13 vs Mibef: *p* < 0.0001). *N* = 3; *n* = Veh: 190; IL-13: 160; CNQX: 197; NBQX: 198; MK801: 234; Mibef: 202 neurons. **g**, **h** Cell specific inhibition of the AMPAR using the YM90K-DART (100 nM) pharmacology tethered to a transmembranal HaloTag (HaloTag-TM) significantly decreases CREB phosphorylation 1 h post IL-13 treatment (50 ng/ml; *p* = 0.0157). *N* = 4; *n* = HT-/IL-13-: 440; HT+/IL-13-: 70; HT-/IL-13+: 322; HT+/IL-13+: 62 neurons. **i**, **j** Cell specific genetic mutagenesis of Grin1 using CRISPR-Cas9/BFP-Cre transfected cells significantly decreases CREB phosphorylation 1 h post IL-13 treatment (50 ng/ml: yellow arrows; *p* < 0.0001). *N* = 5; *n* = BFP-/IL-13-: 324; BFP+/IL-13-: 77; BFP-/IL-13+: 366; BFP+/IL-13+: 46 neurons. Vehicle (0.1% DMSO) was used as control in (**a**–**f**); (0.1% BSA) was used as control in (**g**–**i**). Scale bar: 20 μm. **: *p* < 0.01; ***: *p* < 0.001; ****: *p* < 0.0001. **b**, **d**, **f**, **h**, **j** One-way ANOVA with Sidak’s multiple comparison. Source data are provided as a Source Data file.

Our array screening identified a substantial number of signaling cascades set in motion by IL-13 and several substrates (in particular, several NMDA and AMPA glutamate receptors). To (i) validate the engagement of signaling kinases and (ii) determine their involvement in transcriptional responses evoked by IL-13, we performed a small-molecule inhibitor screening, using CREB phosphorylation as a readout. IL-13-induced phosphorylation of CREB was completely prevented by the JAK inhibitor Ruxolitinib and by the ERK inhibitor PD98059, in agreement with their role in proximal signaling events upon IL-13Ra1 engagement (Fig. 5a, b). Among the downstream signaling molecules identified by the array, we then verified the role of CDK5, GSK-3β, CaMKII and PKA (using Roscovitine, CHIR, KN-93 and H89); we also studied the effects of a TrkB inhibitor (Fig. 5c, d), ANA-12 (since TrkB appears among the up-phosphorylated proteins in the proteome, showed in Fig. 3a, b). IL-13-induced phosphorylation of CREB was prevented by PKA and CaMKII inhibitors, but not by CDK5 or GSK-3β inhibitors (Fig. 5c, d and Supplementary Fig. 6a) Interestingly, ANA-12 partially decreased the effect of IL-13, indicating some degree of involvement of BDNF on IL-13 signaling.

Since CaMKII and PKA cascades are strongly activated by glutamate receptors, and glutamate receptors are prominently represented among the targets of IL-13, we hypothesized that IL-13-driven CREB phosphorylation may be ultimately dependent on glutamate receptor function. We used two AMPAR antagonists (NBQX and CNQX), an NMDAR antagonist (MK-801) and a Voltage-dependent Calcium Channel inhibitor (Mibefradil) to verify our hypothesis. Indeed, IL-13-induced CREB phosphorylation was substantially diminished by AMPAR antagonists NBQX and CNQX as well as by Mibefradil but was completely abolished by the NMDAR antagonist MK-801 (Fig. 5e, f).

We further confirmed the role of postsynaptic AMPAR by DART pharmacology^32^. Cultured neurons were transfected with transmembrane HaloTag-tdTomato (HaloTag-TM; HT) and pre-treated with a chloroalkane-linked AMPAR antagonist (YM90K-DART); the covalent binding of the YM90K-DART to HT highly enriches the local concentration of the antagonist and generates the preferential blockade of AMPAR on the transfected cell. We found that, while untransfected neurons showed the predicted increase in CREB phosphorylation 1 h after IL-13 treatment, HT+ did not, confirming that postsynaptic AMPAR is necessary for IL-13 (Fig. 5g, h). Furthermore, we employed CRISPR-Cas9 to knock-down the expression of the NR1 subunit to confirm the role of NMDAR in IL-13 effects. Cortical neurons were co-transfected with a plasmid encoding BFP-Cre^33^ and with a plasmid encoding the Cre dependent CRISPR-Cas-Grin1 plasmid^34^. BFP + neurons displayed a significant decrease in NMDAR-NR1(GluN1) expression in both the vehicle and IL-13 treatment group (Supplementary Fig. 6b, c). Notably, whereas BFP- cells revealed a significant increase in CREB phosphorylation 1 h after IL-13 treatment, BFP + cells did not (Fig. 5i, j), confirming the role of NMDAR in IL-13 effects.

In conclusion, IL-13 activates its cognate receptor IL-13Ra1 at synaptic sites and sets in motion signaling events involving JAK and ERK, as well as NMDA and AMPA glutamate receptors and CaMKII and PKA, ultimately leading to CREB, STAT3 and STAT6 phosphorylation as well as the induction of multiple IEGs.

### IL-13 upregulates synaptic activity

The increased phosphorylation of glutamate receptors and presynaptic proteins, together with the increase in pCREB and the induction of multiple IEGs, provide converging support to the hypothesis that the ultimate effect of IL-13 is to increase synaptic activity.

**Fig. 6:**
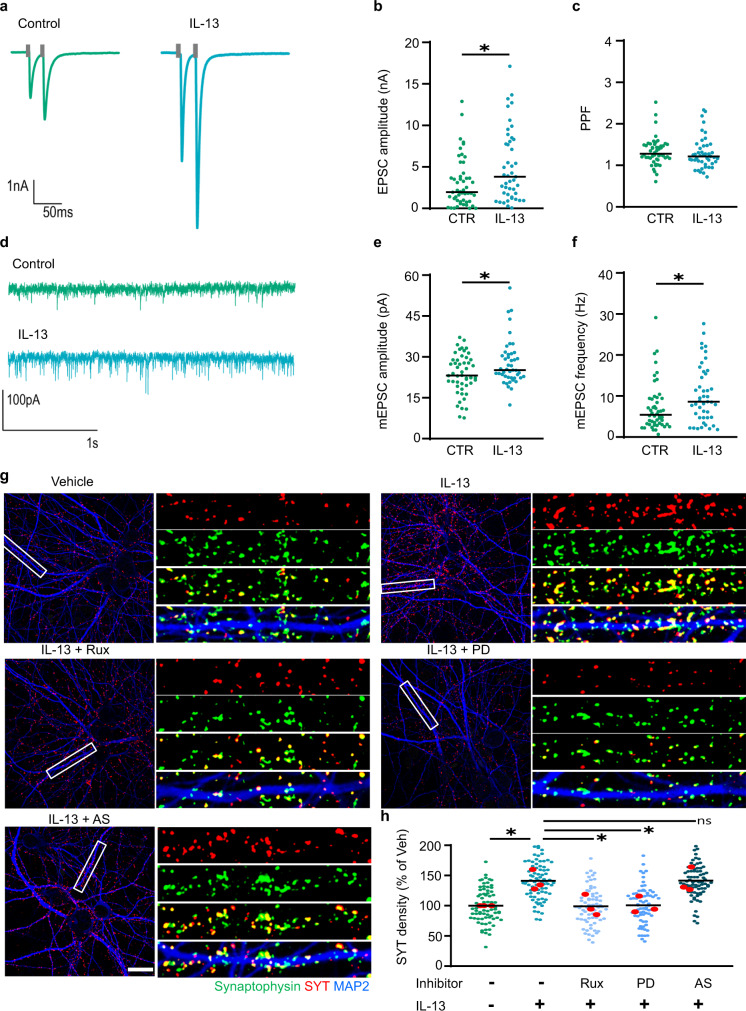
IL-13 increases synaptic activity. **a**, **b** Significant increase in the amplitude of excitatory postsynaptic currents (EPSC) after 30 min of IL-13 treatment (*p* = 0.0139). *N* = CTR: 50 cells; IL-13: 45 cells. **c** No significant difference in paired pulse facilitation (PPF - interstimulus interval 25 ms) after 30 min of IL-13 treatment (*p* = 0.2508). *N* = CTR: 50 cells; IL-13: 45 cells. **d**, **e** Significant increase in the amplitude of miniature excitatory postsynaptic currents (mEPSC) after 30 min of IL-13 treatment (*p* = 0.0475). *N* = CTR: 50 cells; IL-13: 45 cells. **f** Significant increase in the frequency of miniature excitatory postsynaptic currents (mEPSC) after 30 min of IL-13 treatment (*p* = 0.0179). *N* = CTR: 50 cells; IL-13: 45 cells. **g**, **h** Anti-synaptotagmin antibody feeding assay; significant increase of anti-synaptotagmin-labelled synapses after IL-13 treatment in rat cortical neurons. JAK (Ruxolitinib; 280 nM) and ERK1/2 (PD98059; 20 μM) inhibitors significantly abolish the IL-13 induced synaptotagmin labelling (veh vs IL-13: *p* = 0.0366; IL-13 vs Rux: *p* = 0.0344; IL-13 vs PD: *p* = 0.0368). STAT6 (AS1517499; 1 μM) inhibitor did not alter IL-13 induced synaptotagmin labelling (*p* > 0.9999). Total number of synapses was assessed by synaptophysin immunostaining after fixation/permeabilization. *N* = 3; *n* = Veh: 72; IL-13: 78; Rux: 69; PD: 69; AS: 76 dendrites. IL-13 used at 50 ng/mL; 0.1% BSA (electrophysiology) or 0.1% DMSO (pharmacology) as vehicle control. Scale bar overview: 20 μm, insert: 10 μm. *: *p* < 0.05. **b**, **c**, **e**, **f** two-tailed Mann–Whitney test. **h** One-way ANOVA with Sidak’s multiple comparison. Source data are provided as a Source Data file.

We exploited electrophysiological recordings from mouse hippocampal neuron autapses^35^ to investigate this point. IL-13 treatment significantly increased the amplitude of excitatory postsynaptic currents EPSC (EPSC; Fig. 6a, b), confirming the synaptic effects of IL-13. To distinguish pre- and postsynaptic effects of IL-13, we considered the frequency and the amplitude of miniature EPSC. In agreement with the increased synaptic insertion of glutamate receptors, mEPSC amplitude was significantly increased by IL-13 (Fig. 6d, e). Interestingly, we also observed a significant increase in the frequency of mEPSC (Fig. 6f), suggesting an effect of IL-13 on the synaptic vesicle-release process (in agreement with the up-phosphorylation of presynaptic proteins).

Next, we used the anti-synaptotagmin feeding assay^36^ to independently assess the rate of synaptic activity. In this assay, an antibody against an endoluminal epitope of synaptotagmin was added to the culture medium, so that only the synapses with an active cycling of presynaptic vesicles (where the synaptotagmin luminal epitope is exposed to the external medium before being re-endocytosed) are labelled. Therefore, the anti-synaptotagmin antibody labelled the active synapses, and a synaptophysin co-staining was used to assess the total number of synapses. Most importantly, IL-13 treatment significantly increased the ratio of active synaptic terminals after 1 h of treatment (Fig. 6g, h). Interestingly, this effect was blocked by JAK and ERK but not by STAT6 inhibitors, confirming the increased synaptic activity triggered by IL-13 and the related signaling pathways (Fig. 6g, h).

### IL-13 is upregulated upon trauma through activity-dependent and nuclear-calcium-regulated transcription

If IL-13 is a neuron-derived regulator of synaptic function, how is its expression regulated?

In physiological conditions, STAT6 is largely dispensable for *IL-13* induction in the brain, since in *STAT6*^*−/−*^ mice the levels of IL-13 protein in the brain are decreased by approximately 17% compared to WT littermates (Supplementary Fig. 7a–c).

We verified that, as previously reported^8^, traumatic brain injury (TBI) resulted in the upregulation of *IL-13* mRNA in both VGLUT1+ cells in Layer II/III and, though to a lesser extent, in VGLUT2 + cells in layer IV (Supplementary Fig. 7d–h).

**Fig. 7:**
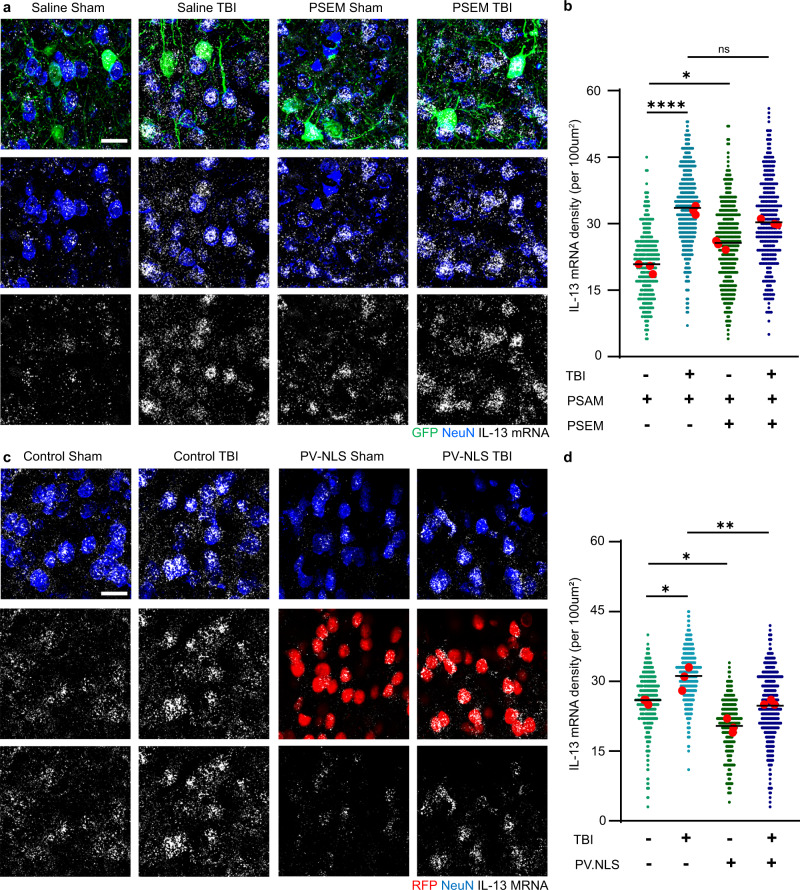
Upregulation of neuronal *IL-13* is driven by neuronal activity and nuclear calcium signaling upon traumatic brain injury. **a**, **b** Upregulation of *IL-13* mRNA expression upon blunt, closed TBI in mouse (3 h post injury). Chemogenetic suppression of inhibitory PV interneurons enhances the upregulation of *IL-13* mRNA at baseline and after trauma (Saline Sham vs Saline TBI: *p* < 0.0001; Saline Sham vs PSEM Sham: *p* = 0.0150; Saline TBI vs PSEM TBI: *p* = 0.0742). *N* = 3; *n* = SS: 332; ST: 422; PS: 382; PT: 319 neurons. **c**, **d** Neuronal nuclear calcium buffering (PV.NLS) strongly reduces *IL-13* mRNA at baseline and upon TBI (Control Sham vs Control TBI: *p* = 0.0141; Control Sham vs PV.NLS Sham: *p* = 0.00141; Control TBI vs PV.NLS TBI: *p* = 0.0069). *N* = 3; *n* = MS: 427; MT: 394; PS: 215; PT: 385 neurons. Scale bar: 20 μm. *: *p* < 0.05; **: *p* < 0.01; ****: *p* < 0.0001. **b**, **d** One-way ANOVA with Sidak’s multiple comparison. Source data are provided as a Source Data file.

We explored in vivo whether the induction of *IL-13* could be dependent on neuronal activity; we used a chemogenetic anion-permeable ion channel (PSAM/PSEM system^37^) to inactivate Parvalbumin-positive interneurons, to increase neuronal firing. We performed this manipulation in healthy conditions as well as in the context of acute TBI. Injection of the AAV9 in the PV-Cre mice resulted in >95% of PV interneurons expressing the inhibitory PSAM. Silencing PV interneurons (by PSAM expression and PSEM administration) resulted in a significant increase in *IL-13* mRNA in neurons compared to control mice (expressing PSAM but administered only the vehicle). Interestingly, TBI alone also upregulated neuronal *IL-13* expression, which was not further upregulated by the concomitant chemogenetic silencing of PV interneurons (Fig. 7a, b). Thus, increased neuronal activity resulted in enhanced *IL-13* transcription at baseline and upon TBI.

To verify this hypothesis, we set out to buffer the nuclear Ca^2+^ signals that are involved in activity-dependent transcription^38^. We exploited the PV-NLS-mCherry construct (previously described^39^), in which the Ca^2+^-binding protein Parvalbumin is targeted to the nucleus by an attached Nuclear Localization Signal (together with the mCherry tag). The construct was expressed under hSyn promoter and delivered via injection of AAV9 (as control, an empty vector was used).

In sham mice, expression of PV-NLS significantly reduced *IL-13* expression compared to the empty-vector. Furthermore, whereas in empty vector-injected mice TBI resulted in the substantial elevation in *IL-13* expression, PV-NLS completely blocked this effect. Thus, baseline expression of *IL-13* is linked to neuronal activity and nuclear Ca^2+^ signaling, and *IL-13* upregulation upon TBI is enhanced by increased neuronal excitability but suppressed by the blunting of nuclear Ca^2+^ signaling (Fig. 7c, d).

### IL-13 reduces neuronal sensitivity to excitotoxic cell death

**Fig. 8:**
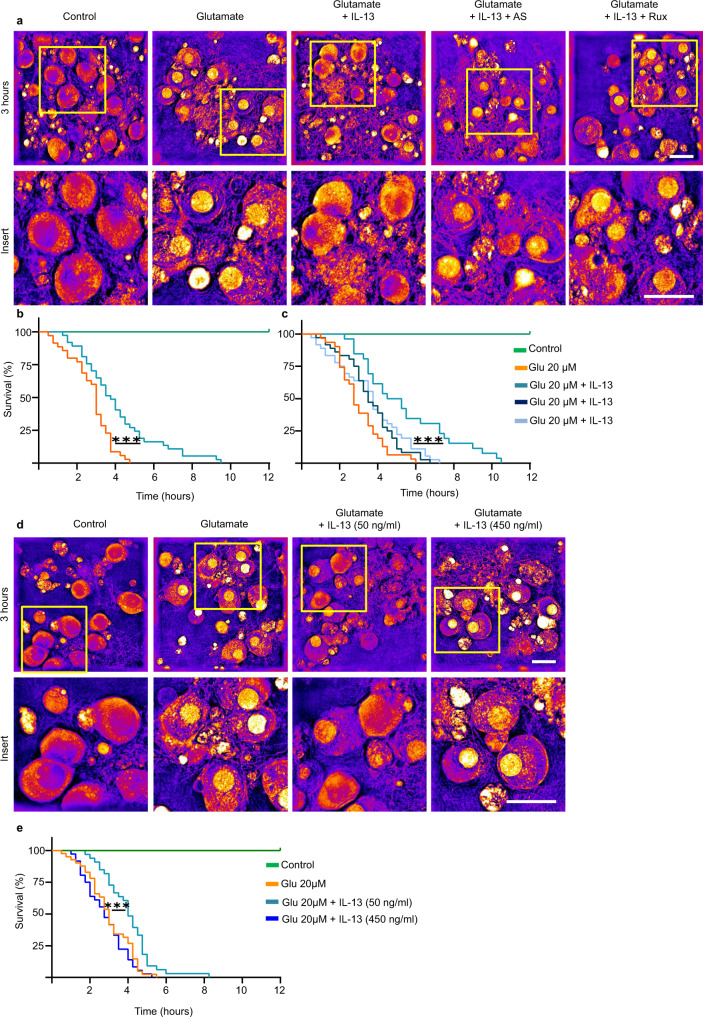
IL-13 reduces excitotoxic neuronal death. **a**–**c** Significant reduction of glutamate (20 μM) induced neuronal toxicity after IL-13 treatment (50 ng/ml) in rat cortical neurons revealed in holotomography microscopy live imaging (*p* = 0.0003). Treatment with JAK (Ruxolitinib; 280 nM) and STAT6 (AS1517499; 1 μM) inhibitors show that the protective effects of IL-13 are dependent on a JAK/STAT6 dependent mechanism (IL-13 vs Rux: *p* = 0.0030; IL-13 vs AS: *p* = 0.0006). *N* = 4; *n* = C: 40; 20 μM: 35; 20 μM + IL-13: 37; AS: 36; Rux: 36 neurons. **d**, **e** High dose IL-13 (450 ng/ml) does not prevent to glutamate induced neuronal toxicity (IL-13 450 ng/ml vs IL-13 50 ng/ml: *p* = 0.0002). *N* = 4; *n* = C: 41; 20 μM: 41; 20 μM + IL-13 50 ng/ml: 33; 20 μM + IL-13 450 ng/ml: 36 neurons. 0.1% BSA + 0.1% DMSO used as vehicle control. Scale bar: 20 μm. **: *p* < 0.01; ***: *p* < 0.001. **b**, **c**, **e** Log-rank (Mantel–Cox) test. Source data are provided as a Source Data file.

What is the ultimate impact of IL-13 upregulation upon TBI, on neuronal vulnerability? The increase in CREB phosphorylation suggests that IL-13 may be beneficial for the cell; conversely, detrimental effects of IL-13 have also been proposed^11^. We set out to test this hypothesis in an experimentally tractable system. We used a holotomographic microscope^40^ to obtain high-contrast, label-free, time-lapsed imaging of cultured cortical neurons for 12 h, acquiring a three-dimensional holographic stack every 15 min. Neuronal cultures were exposed to vehicles or to 20 µM or 40 µM glutamate either alone to simulate an acute excitotoxic environment associated with traumatic injury, or in the presence of IL-13 (50 ng/ml; added simultaneously to glutamate). We monitored individual cells for the appearance of nuclear pyknosis (nuclear condensation with substantial increase in refractive index) as a sign of cell sufferance and death; data were recorded for each cell at the timepoint when nuclear pyknosis plateaued. In vehicle-co-treated cultures, exposure to glutamate (20 or 40 µM) caused a rapid and relentless increase in nuclear condensation, which plateaued between 3 and 4.5 h after the exposure; no nuclear pyknosis was observed in vehicle-treated cells (Fig. 8a, b and Supplementary Fig. 8a, b). Remarkably, co-treatment with IL-13 significantly improved the survival of cultured neurons exposed to either 20 or 40 µM glutamate, with median time to pyknosis prolonged from 5 to 9.5 h (Fig. 8a, b and Supplementary Fig. 8a, b). Interestingly, the beneficial effect of IL-13 was abolished by the co-treatment with inhibitors of IL-13 signaling such as the JAK inhibitor Ruxolitinib as well as the STAT6 inhibitor AS1517499 (Fig. 8a–c). In line with the dose-dependent effects on the phosphorylation of CREB, the beneficial effects of IL-13 on neuronal survival appear to be restricted to lower concentrations of IL-13. In fact, the high dose of IL-13 (450 ng/ml) which was unable to increase pCREB levels did not ameliorate neuronal survival upon a glutamate excitotoxicity challenge (Fig. 8d, e).

### IL-13 and IL-13Ra1 are expressed in human neurons and are upregulated in brain and CSF of TBI patients

Using three distinct approaches, we addressed the relevance of our murine investigations of IL-13 in normal human brain and in neurotrauma patients.

**Fig. 9:**
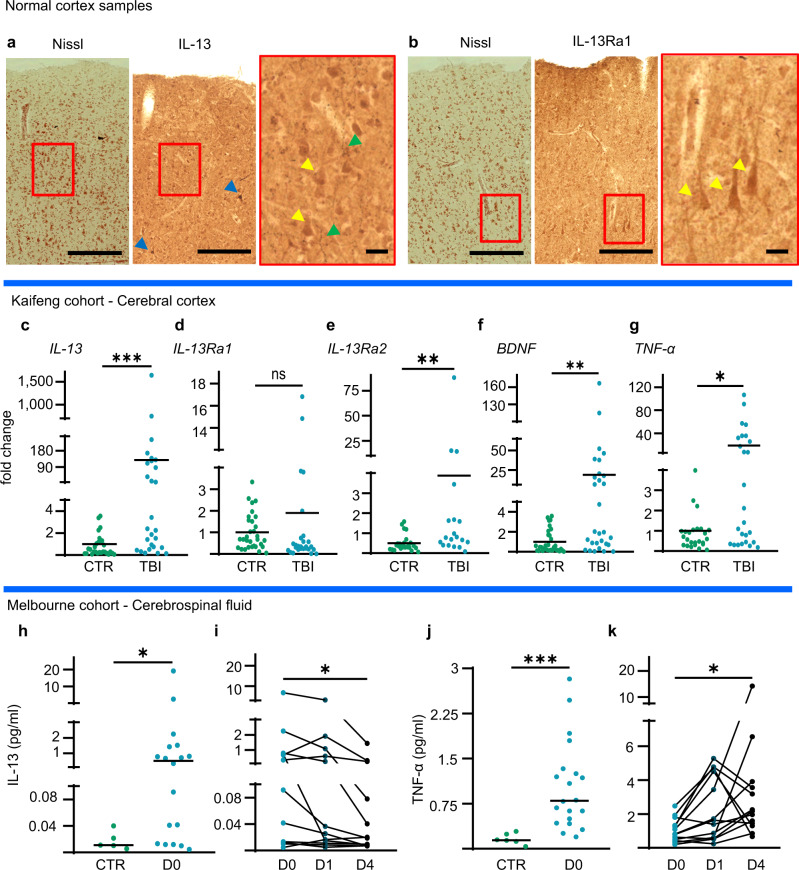
IL-13 is expressed in the human brain and is upregulated upon TBI in the human cortex and CSF. **a** Darrow red Pigment-Nissl and Immunohistochemistry for IL-13 shows moderate, high and very high-expressing neuronal populations in human post-mortem cortical tissue. *N* = 3. **b** Darrow red Pigment-Nissl and Immunohistochemical staining of IL-13Ra1 shows a large number of neurons across all cortical layers of post-mortem cortical tissue. *N* = 3. **a**, **b** Scale bar overview: 100 μm, scale bar insert: 10 μm. **c**–**g** Significant upregulation of the mRNA for *IL-13* (*p* = 0.0002), *IL-13Ra2* (*p* = 0.0021), *BDNF* (*p* = 0.0015) and *TNF-α* (*p* = 0.0160), but not of *IL-13Ra1* (*p* = 0.0998), in human cortical tissue samples resected after traumatic brain injury vs controls from elective surgery. (RT-qPCR). CTR *N* = 34; TBI *N* = 29. **h** Significant upregulation of IL-13 protein in cerebrospinal fluid samples from severe TBI patients within 24 h of TBI (*p* = 0.0243). CTR *N* = 5; TBI *N* = 18. **i** Time course of CSF levels of IL-13 in severe TBI patients: progressive decrease of IL-13 levels between D0 and D4 (*p* = 0.0016). D0 *N* = 12; D1 *N* = 12; D4 *N* = 12. **j** Significant upregulation of TNF- α in CSF of TBI patients (*p* < 0.0001). CTR *N* = 5; TBI *N* = 20. **k** Progressive increase of TNF-α levels in the CSF of severe TBI patients between d0 and D4 (*p* = 0.0181). D0 *N* = 12; D1 *N* = 12; D4 *N* = 12.Cytokines measured by SIMOA assay. *: *p* < 0.05. **: *p* < 0.01. ***: *p* < 0.001. **c**–**g**, **h**, **j** Two-tailed Mann–Whitney test. **i**, **k** Friedman test with Dunn’s multiple comparison. Source data are provided as a Source Data file.

Firstly, we explored the immunoreactivity for IL-13 and IL-13Ra1 in samples of motor cortex from four independent healthy (i.e., not affected by trauma or neurodegenerative processes, full details are reported in Supplementary Table 1) post-mortem human brains. To highlight the cellular architecture and neuronal distribution, we performed a Darrow red Pigment-Nissl stain on sections consecutive to those used for the immunohistochemistry. In absence of primary antibodies, no immunohistochemical reactivity was observed (negative control, Supplementary Fig. 9a). IL-13 immunoreactivity was detected in two populations: (i) moderately positive cells, with morphology and Nissl-staining compatible with neuronal identity, were seen across cortical layers, in particular in the upper layers and immunoreactivity concentrated in the cell body and proximal dendrites (Fig. 9a1) and (ii) a comparatively smaller number of cells with neuronal morphology displayed a strong IL-13 immunoreactivity (Fig. 9a1 and Supplementary Fig. 9b). Across the neuropil, a punctate IL-13 immunoreactivity with overall bead-string morphology was observed (green arrowheads, Fig. 9a1 and Supplementary Fig. 9b). On the other hand, IL-13Ra1 expression appeared more homogeneous in terms of expression intensity, with a large number of cells with neuronal morphology showing iIL-13Ra1 immunoreactivity the cell body and the dendrites (Fig. 9b1-2 and Supplementary Fig. 9c).

Altogether, our experiments show that the human brain displays a pattern of IL-13 and IL-13Ra1 expression compatible with that seen in the murine brain, validating our experimental models.

Secondly, we investigated whether the upregulation of IL-13 transcription observed in murine models could be confirmed in human samples. Human cortical samples (*n* = 29) resected during the neurosurgical treatment of acute TBI and, as controls, fragments of healthy human cortex obtained during elective neurosurgery (clipping of unruptured aneurysms, *n* = 34; full details of the two groups -collectively henceforth defined as “Kaifeng Cohort”- are reported in Supplementary Table 2) were processed to extract the total RNA content for assessment of gene expression. Overall, TBI samples displayed a significantly higher level of *IL-13* expression compared to control cortical samples (Fig. 9c), in agreement with the observations in the murine model. Interestingly, the expression of *IL-13Ra1* was unchanged in TBI samples (Fig. 9d), whereas *IL-13Ra2* was upregulated upon TBI (Fig. 9e). The expression of the inflammatory marker *TNF-α* and of the neurotrophin *BDNF* was also upregulated after TBI (Fig. 9f, g).

Thirdly, we explored whether the elevation of IL-13 could be replicated in the cerebrospinal fluid (CSF) of moderate-severe TBI patients. We analyzed samples obtained from CSF drainage from TBI patients (day 0 *n* = 18, day 1 *n* = 27, day 4 *n* = 28), or, as controls, from patients subjected to CSF drainage for non-traumatic reasons (*n* = 6). This cohort (henceforth collectively defined as “Melbourne Cohort”) is independent of the Kaifeng Cohort and has been the object of publication before^41^; clinical and demographic characteristics are briefly summarized in Supplementary Table 3. We used a Single-Molecule Array (SIMOA) platform to determine the concentration of IL-13 and, as a proxy of inflammation^42^, of TNF-α in CSF samples obtained within 24 h of TBI (comparably to the Kaifeng Cohort); for a distinct subset of patients. Serial samples were available from day 0 (within 24 h of trauma), day 1 and day 4 and these samples were independently analyzed. We found that, compared to controls, TBI patients displayed a very strong elevation, although with substantial variability from case to case, in the level of IL-13 in the acute phase of TBI (<24 h; Fig. 9h), in agreement with the murine data and the Kaifeng cohort expression data. The analysis of the serial samples showed that in most cases IL-13 levels tended to peak already at day 0 and then progressively declined over time (day 0 vs day 4; Fig. 9i). Although TNF-α was also strongly elevated upon TBI (Fig. 9j), there was no correlation between IL-13 and TNF-α levels with TNF-α levels peaking (in all but 2 cases) at day 1 or later (Fig. 9k).

Taken together, the findings from the Kaifeng Cohort and the Melbourne Cohort are in remarkable agreement and demonstrate that upon trauma the cortex and the CSF display a massive but transient increase in IL-13 levels, which are largely uncorrelated with the upregulation of inflammatory cytokines. Therefore, human neurons are exposed to increased levels of IL-13, making our experimental investigation in murine models relevant to human TBI.

## Discussion

In the present study, we provide convincing evidence pointing toward a previously unappreciated role of IL-13 as an endogenous regulator of neuronal origin, synaptic biology, and neuronal activity. Furthermore, we show that elevation of IL-13 is a common phenomenon that takes place in rodents as well as human brains, and that in the condition of brain injury, IL-13 has fundamentally beneficial effects in protecting neuronal survival.

Our data support the notion that IL-13 neuronal biology shares several similarities with synaptic modulators such as neurotrophins. In fact, *IL-13* is upregulated upon increased neuronal firing at baseline and in TBI, and its neural transcription is loosely dependent on STAT6 (in contrast to immune cells) but strongly dependent upon nuclear-calcium signals^38^. Furthermore, IL-13 causes a significant increase in NMDA and AMPA receptor phosphorylation, both events associated with the increased recruitment of these glutamate receptors at the synapses and their trafficking to the surface^43–45^. In addition, the upregulation of synaptic activity and glutamate receptor activity results in a dramatic elevation of CREB phosphorylation and, in turn, transcription of several CREB-regulated genes. Taken together, these data suggest that IL-13 is a previously unknown mediator of synaptic plasticity, displaying activity-dependent potentiation and stabilization (summarized in Supplementary Fig. 10). On this ground, it can be hypothesized that loss of IL-13 may produce phenotypes associated with reduced learning and impaired memory retention.

In support of this hypothesis, *IL-13*^*−/−*^ mice do display abnormalities often related to disturbances of synaptic plasticity: in fact, *IL-13*^*−/−*^ mice performed very poorly in a 4-day Morris Water Maze (MWM) test and almost completely failed at reversal learning in the same setting^12^. Interestingly, similar, although less prominent, behavioral abnormalities were observed in mice lacking the IL-4 and IL-13 co-receptor (*IL-4Ra*^*−/−*^ mice); these mice displayed significantly longer latencies to locate the platform in the MWM test. Although these abnormalities were attributed to the impairment of BDNF secretion by astrocytes under the control of adaptive immunity cells^12^, we propose that failure in synaptic plasticity due to the loss of neuronal IL-13 may substantially contribute to this phenotype. In fact, IL-13 appears to drive phosphorylation events associated with the increased insertion in synapses and increased neuronal activity, both recognized as the hallmarks of the learning process. In this context, additional mechanisms may be involved but remain to be investigated. It could be assumed that IL-13 produced by neurons may reach local microglia through spill-over and may induce a specific microglial phenotype^46^ involved in the remodeling of synaptic contacts^1^.

Our data also show that neurons exposed to IL-13 display a reduced sensitivity to excitotoxic cell death. At least two mechanisms may be responsible for this effect: firstly, IL-13 could upregulate synaptic signaling leading to CREB phosphorylation, a process well-known to be associated with neuroprotection and reduced neuronal vulnerability^47,48^; secondly, after the initial upregulation, IL-13 could lead to a rapid de-phosphorylation of many NMDA and AMPA receptor subunits, conducive to their removal from cell surface and thus effectively blocking glutamate-dependent ion fluxes. Recently, additional mechanisms for beneficial properties of IL-13 in pathological conditions have been proposed, with a major focus on the neuroimmunological aspects. In particular, insertion of cells providing a continuous source of IL-13 resulted in a strong M2 polarization of microglia and macrophages upon stroke^49^. A similar shift was observed also upon peripheral administration of IL-13^50^. However, it must be noted that in a severe, permanent carotid artery occlusion model of stroke, simultaneous deletion of IL-13, IL-9, IL-4, and IL-5 did not worsen the neurological outcome^51^. In the context of TBI, IL-13 has been shown to attenuate the acute motor deficits in the rotarod test and produce a faster recovery in the foot-fall and wire-hanging tests when administered through intranasal instillation. A decrease in the so-called pro-inflammatory microglial phenotype together with an increased microglial phagocytosis is thought to be involved in these beneficial effects^52^. The anti-inflammatory properties of IL-13 have been also associated with its ability to trigger the apoptosis of reactive microglia^53,54^. However, in an experimental model of TBI (controlled cortical injury), suppression of IL-13 through the administration of a neutralizing antibody protected neurons from the induction of piroptosis^55^. Concordant with this detrimental function, it was reported that IL-13 increased oxygen radical (ROS) production by dopaminergic neurons^56,57^ and that ablation of neuronal IL-13 signaling (in IL-13Ra1 knock-outs) prevented the loss of dopaminergic neurons in the substantia nigra under chronic stress^11^. Our findings help to reconcile this conflicting evidence around the role of IL-13: in fact, increased phosphorylation of glutamate receptors and their enhanced membrane localization may amplify the excitatory inputs which, depending on the neuronal type and the concentration, may enhance ROS production and, at the same time, trigger CREB-dependent transcriptional responses. In this regard, here we show that the impact of IL-13 on CREB phosphorylation is not linear but inverse-U-shaped: so, the increase or decrease in CREB activation may depend on the amount of IL-13 available and the resulting cellular response. We speculate that while at lower concentrations IL-13 may promote the signaling through synaptic NMDAR, higher concentrations may simulate the insertion of extrasynaptic NMDAR whose distinct signaling may result in the CREB shut-down^47,58^.

In summary, we have used three distinct approaches to demonstrate the relevance of our findings on the role of IL-13 for human TBI. We define IL-13 and IL-13Ra1 immunoreactivity in at least two neuronal populations in the normal human cortex, whose level of expression differed significantly. Since immunostaining in human post-mortem tissue is subject to potential artifacts, it is not possible to conclude that these are indeed distinct cell populations; however, neuronal immunoreactivity for IL-13 and IL-13Ra1 does confirm that murine findings recapitulate at least some aspects of the human biology. The analysis of IL-13 expression in CSF and brain samples from TBI patients further confirms that the upregulation of IL-13 observed in mice does take place in humans as well. Nevertheless, some limitations apply to the use of human tissues. For the histology on normal human cortex, double-immunostaining to confirm neuronal identity was prevented by the over-fixation due to the prolonged storage in PFA. Regarding the surgical samples, we found that some samples had small amounts of a variety of mRNAs, suggesting the presence of necrotic tissue. In contrast, IL-13 concentrations did not correlate with TNF-α, further indicating that IL-13 upregulation is not a direct consequence of inflammatory responses to TBI. Interestingly, the findings from the Kaifeng Cohort were corroborated by the Melbourne cohort, where, once again, IL-13 was found elevated (in agreement with previous reports^59^), however without any associations with the levels of TNF-α in CSF. As shown previously for other cytokines measured in the Melbourne cohort^41^, significant variability was observed in the concentrations of IL-13 in CSF, which ranged from control levels to almost 100-fold the baseline. Since the Melbourne cohort includes patients with substantial variability in TBI classification, extent of brain damage and prognosis^41^, it can be postulated that IL-13 elevation may be characteristic to a subset of patients. In this respect, the role of IL-13 in patient stratification and prognosis is still unresolved and may need a substantially larger cohort size to be established. Nevertheless, the findings of the Kaifeng and Melbourne patient cohorts indicate that human neurons are exposed to high levels of IL-13 upon TBI, both at the site of injury and in the CSF. It is worth noting that the experimental murine TBI paradigm employed here produced a less severe injury (Neurological Severity Score 0–1, see methods) than those experienced by the human patients; this is because mild TBI human subjects do not undergo neurosurgical intervention or CSF shunting. Nevertheless, the mild murine TBI data and the human data are in general agreement, suggesting that IL-13 upregulation is a phenomenon shared across species and across degrees of injury severity. Therefore, the investigation of the effects of neuronal exposure to IL-13 in in vitro or murine models has translational validity.

The evidence brought forward in this study, identifying IL-13 as a neuronal regulator of synaptic structure and function, will certainly pave the way to explore several unresolved questions around the role of IL-13 in brain physiology and pathophysiology. This is especially relevant in the light of anti-IL-13 therapeutics being developed for allergic conditions as well as against gliomas^60^.

Dysregulation of synaptic plasticity and microglial reactivity are two aspects often encountered in acute and chronic neurological and psychiatric conditions^61^. Our findings reveal that IL-13 may be one part of the machinery involved in both systems and may provide new entry points for therapeutic manipulation of both at once.

## Methods

### Animals

Primary neuronal rat cultures were approved by the Ulm University veterinary and animal experimentation committee under license number O.103-12. Intracerebral AAV injection, chemogenetics and TBI were approved by the Regierungspräsidium Tübingen under license number 1420.

*STAT6*^*−/−*^ mice (B6.129S2(C)-*Stat6*^*tm1Gru*^/J) were previously described^62^ and were backcrossed for more than ten generations to BALB/c background^63^. *IL-13*^*−/−*^ mice (Il13t^m1.1Anjm^) were previously reported^25^ and have been bred on a C57/BL6J genetic background for >10 generations^64^; *IL-13*^*−/*−^ mice were housed in the Biomedical Resource Center at the Medical College of Wisconsin, an AAALAC-approved facility. All protocols in this study were approved by the American Association for Laboratory Animal Science (IACUC) and local Animal Care and Use Committee (AUA00005516).

PV-Cre mice (B6.129P2-*Pvalb*^*tm1(cre)Arbr*^/J), a kind gift of Silvia Arber and Pico Caroni, were used for the in vivo TBI experiments and the chemogenetic experiments.

For histology and western blot on normal mouse cortex, WT mice (B6SJLF1/J) were used. All experiments were performed on male adult mice (p50-60). All mice were group housed from weaning to the time of the procedure, with ad-libitum access to food and water, and under a light/dark cycle of 12/12 h, at 24 °C and humidity 60–80%.

### Mouse model of traumatic brain injury

Traumatic brain injury was induced using a modified closed weight drop model^65^ delivered using a stereotactic apparatus^66^. Briefly, adult mice aged p60-80 were anesthetized with 5% sevoflurane in 95% O_2_ and treated with 0.1 mg/Kg of buprenorphine prior to the injury. The skin was incised on the midline to expose the skull and the mouse was positioned in the weight drop apparatus, in which the head was fixed onto the holding frame. TBI was delivered by dropping a weight of 120 g from a height of 40 cm with a displacement into the skull of 1.5 mm onto the coordinates of the injection site (x = +2.0; y = −2.0; z = 0.5). 100% of O_2_ was administered after the impact until normal breathing was restored. After the TBI, the skin was sutured with Prolene 6-0 and mice were transferred to their cage with water and food ad-libitum. Mice were subject to neurological examination 3 h after TBI using the Neurological Severity Score standardized assessment (performed as previously reported^67,68^; in agreement with previous reports, all mice had an NSS score not higher than 1, thus defining this as a mild TBI model. This degree of severity was chosen because (i) it is ethically acceptable since it delivers no well-being burden on experimental animals (ii) corresponds to the most common TBI occurrence in human subjects (iii) it is highly reproducible and (iv) it provides an experimentally tractable model, since there is no bone fracture, haematoma or necrotic area, while still displaying neuronal loss, microgliosis and astrogliosis. Mice were then euthanized (3 h post TBI) for further experimentation.

### Immunofluorescence staining on mouse brain

Brain samples were processed as previously described^8^. Briefly, mice were euthanised with a lethal dose of a mixture of ketamine (100 mg/kg) and xylazine (16 mg/kg) in PBS followed by perfusion with 4% PFA in PBS, brain tissue was dissected and post-fixed in 4% PFA overnight. Brain tissue was washed and cryoprotected in 30% sucrose for 2 days, after which the samples were embedded in OCT (Tissue Tek). 40 µm sections were cut and subsequently blocked (3% BSA, 0.3% triton-X100, 1× PBS) for 2 h at room temperature (RT), after which primary antibodies (Supplementary Table 4) were diluted in blocking buffer and incubated for 48 h at 4 °C. Sections were washed 3 × 30 min followed by incubation with secondary antibodies (Supplementary Table 5) diluted in blocking buffer for 2 h at RT. The sections were washed 3 × 30 min and mounted with Prolong antifade mounting medium (Invitrogen).

### Single-molecule mRNA in situ hybridization

We performed the single-molecule mRNA in situ hybridization as previously reported^69^ in agreement with the manufacturer’s instructions, (ACDbio, RNAscope, fluorescence in situ mRNA hybridization for fixed frozen tissue sections, all reagents/buffers were provided by ACDbio), with minor adjustments^8^. The detailed protocol is reported in the Supplementary Methods file.

### Dissociated cortical neuronal culture, immunocytochemistry and pharmacological treatments

Primary rat cortical culture was performed as previously reported^36^. Details of the protocol are reported in the Supplementary Methods file.

Immunofluorescence staining was performed as previously described^8^. A detailed protocol is reported in the Supplementary Methods file.

All treatments and experiments were performed on DIV 14-21. Primary cells were treated with IL-13 at 50 ng/ml (for the dose response curve, cells were treated with 5, 15, 50, 150 and 450 ng/ml) for 1 h or 3 h. The inhibitors of transcription factors, ERK inhibitor PD98059 (20 µM, Promega), STAT6 inhibitor AS1517499 (1 µM, Axon Medchem), STAT3 inhibitor Stattic (20 µM, Selleckchem), and JAK inhibitor Ruxolitinib (280 nM, Cayman) were added to the culture medium half an hour before IL-13 exposure. In addition, for pretreatment with the inhibitors of glutamate receptors and calcium channels, primary cultures were exposed to AMPA and kainate receptor blocker CNQX (10 µM, Cayman), AMPA receptor blocker NBQX (10 µM, Cayman), NMDA blocker MK-801 (10 µM, Cayman), and calcium channel blocker Mibefradil (10 µM, Cayman) for half an hour before IL-13 treatment. Similarly, the TrkB receptor antagonist ANA-12 (10 µM, Tocris), PKA inhibitor H89 (10 µM, Tocris), CAMKII inhibitor KN-93 (10 µM, Tocris) CDK5 inhibitor (Roscovitine, 10 µM) and GSK-3β inhibitor CHIR 98014 (10 µM, Tocris) were added 30 min prior to IL-13 treatment. For the excitotoxic insult, glutamate (20 or 40 µM) was added to the culture at the same time as IL-13 treatment. A similar volume of only DMSO was added to control wells.

### Antibody feeding assays

We performed the anti-synaptotagmin antibody feeding protocol, as previously reported, to detect the synaptic vesicle release rate^36^. Briefly, a monoclonal antibody (diluted 1:500) directed against a luminal epitope of Synaptotagmin-1 fluorescently labeled with Oyster®550 (105 103C3, Synaptic System) was added to the culture medium 30 min before fixing. For IL-13 and inhibitors exposure experiments, the same protocol as reported above was applied. After being washed twice with DPBS^−^/^−^, the cells were fixed with 4% paraformaldehyde containing 4% sucrose for 10 min. Then the processed primary cells were used for immunofluorescence staining as described above.

The extracellular NMDAR and AMPAR antibody feeding protocol was performed as previously reported^70^. Cells were treated with IL-13 (50 ng/ml) for 1 h or 3 h or vehicle and were treated with an antibody which binds to the extracellular epitope of NMDAR or AMPAR (1:200) diluted in culture medium for 30 min. Cells were washed and fixed in 4% paraformaldehyde containing 4% sucrose for 10 min. Then the processed primary cells were used for immunofluorescence staining as described above.

### Epifluorescence and confocal imaging

Confocal imaging of brain sections was performed with a laser-scanning confocal microscope (Zeiss LSM 980 and LSM 710, Carl Zeiss). For the AAV2-GFP tracer experiment, images were acquired with a 100× (NA 1.4) oil immersion objective in a 1024 × 1024 pixel 16 bit format. A z-stack of 9 optical sections spanning 1.36 μm (step size of 0.17 μm), 1.7 optical zoom, a laser power of 0.4% and a gain of 800 were acquired.

For the in situ hybridization experiment, images were acquired with a 40× (NA 1.3) oil immersion objective in a 1024 × 1024 pixel 12 bit format. A z-stack of 9 optical sections spanning 9 μm (step size of 1 μm), 1.0 optical zoom, a laser power of ~4.5% and a gain of 800 were acquired.

Confocal imaging of dissociated cultures was performed with a laser-scanning confocal microscope (Leica DMi8) and fluorescence microscope (Keyence BZ-X800E). In confocal images, laser power was set in the range of 5–15%.

For the synaptic colocalization experiments, images were acquired with a 63×(NA 1.3) oil immersion objective in a 1024 × 1024 pixel 12-bit format, a z-stack of 3 optical sections spanning 1.05 μm (step size of 0.35 μm) and 3.0 optical zoom.

For the antibody feeding assay experiments, images were acquired with a 63× (NA 1.3) NA oil immersion objective in a 1024 × 1024 pixel 12 bit format, a z-stack of 7 optical sections spanning 3.5 μm (step size of 0.5 μm) and 1.5 optical zoom.

For the transcription factor experiments, images were acquired with a 40× (NA 1.3) oil immersion objective in a 1024 × 1024 pixel 12 bit format, a z-stack of 10 optical sections spanning 5 μm (step size of 0.5 μm) and 1.5 optical zoom.

For the c-fos experiment, images were acquired with an 20× (NA 0.75) objective in a 1024 × 1024 pixel 8 bit format (Keyence), a tile scan of 5 × 5 images with 1.0 optical zoom and an exposure time of 1/3 s was acquired.

Imaging parameters were set to obtain signals from the stained antibody while avoiding saturation. All fluorescent channels were acquired independently to avoid fluorescence cross-bleed. For each treatment, six areas per coverslip were randomly selected for imaging.

### Super-resolution STED imaging

Super-resolution STED imaging was performed on a Stedycon module (Abberior instruments, Germany) fixed to a Zeiss microscope fitted with a 100× (NA 1.4) oil objective. Images were acquired in both confocal and STED modes, imaging parameters were set to obtain signals from the staining while avoiding saturation. For the imaging, Abberior Star 580 and Abberior Star 635 dyes were used, with an excitation laser of 561 nm for the Star 580 and 640 nm for the Star 635 and a STED laser of 775 nm for both channels. The laser power for the excitation laser was set to 20% for Star 580 and 30% for Star 635, the laser power for the STED laser was set to 50.7% for Star 580 and 46.5% for Star 635. The pixel size was set to 25 nm, with a pixel dwell time of 10 µs and line accumulation of 6. A piece of dendrite was randomly selected, and a single optical section was acquired in correspondence with the optical plane with the maximum dendritic diameter. No post-processing has been performed on these images; raw images are depicted in the figures.

### Brain fractionation and Western blot

Brain fractionation was acquired as described previously^23,24^. The detailed protocol is reported in the Supplementary Methods file.

### RNA extraction and qPCR

RNA isolation was performed as described in the manufacturer’s instructions (QIAzol, Qiagen). The detailed protocol is reported in the Supplementary Methods file.

### Phospho-antibody array assay

Neuroscience Phospho Antibody Array was performed as described in the manufacturer’s instructions (Full Moon BioSystems). Further details are reported in the Supplementary Methods. The data generated in this study have been deposited in the Figshare database under accession code 10.6084/m9.figshare.21758552^71^. For array data analysis, the raw intensity values for each phosphorylated epitope were recorded automatedly via Image recorder software or manually using ImageJ software. The raw data files were loaded in R software and the dataset for each array was preliminarily subjected to quality control assessment; outlier identification, data distribution, intra-array and inter-array normalization. Modified linear modeling-based analysis was then applied to the data to identify targets showing a significant increase or decrease in phosphorylation at different timepoints.

### Autaptic cultures

Primary hippocampal neurons from BL6 mice of both sexes were obtained on postnatal day (P) 0-2, as described previously^35^. In brief, astrocyte feeding layers were prepared 1–2 weeks before the seeding of neurons. To obtain primary neurons, hippocampi were dissected and enzymatically treated with 25 units per ml of papain for 45 min at 37 °C, which was followed by mechanical trituration to dissociate individual neurons. For autaptic cultures, neurons were seeded on micro-islet astrocytes and incubated in Neurobasal A media containing 50 μg/ml streptomycin and 50 IU/ml penicillin at 37 °C until experiments were performed between DIV 15 and 20.

### Electrophysiological recordings

Synaptic function was assessed as described previously^35^. Full details are reported in Supplementary Methods.

### Image analysis and 3D reconstruction

The image analysis was performed using the ImageJ software suite (NIH). For the analysis of the brain sections injected with AAV2-GFP, a confocal stack (acquired with a 100× NA 1.4 oil immersion objective, 1.7 optical zoom) of 9 0.17 µm-optical sections was collapsed into a maximum intensity projection; background was subtracted using the rolling-ball method (20 pixels radius). The profile of the GFP+ dendrites was manually traced and random artifacts-free dendrites of a minimal length of 200 µm were selected. Images were thresholded with a minimum/maximum threshold of fluorescence intensity set at 5000/20,000 (16 bit images); IL-13, IL-13Ra1 and VGLUT + puncta were positively identified if their size was >4 pixels; two spots were scored as “colocalized” if their area after thresholding overlapped for >75%. Synapse density was calculated as percentage of positive VGLUT per 100 µm of dendrite. Surface reconstruction has been performed using the Imaris software suite (Bitplane AG, Zürich, CH). GFP + dendrites were marked using the following parameters: surface grain size: 0.0851 µm; diameter of the largest sphere: 0.391 µm; background subtraction threshold 8946.93 (out of 64768) and a filter surface of 100%. IL-13+ and VGLUT + synapses were marked using the following parameters: surface grain size: 0.0851; diameter of the largest sphere: 0.391; background subtraction threshold: 275 (out of 2274); seed point diameter (split touching objects): 0.425; filter seed points (quality): 162 (48%) and a filter surface of 100%.

For the quantification of the images from dissociated neuronal culture, confocal stacks of 3 0.35 µm-optical sections (acquired with a 63× NA 1.3 oil immersion objective, 3 optical zoom) were collapsed into maximum-intensity projections; background was subtracted by rolling-ball method (20 pixels-radius). Images were thresholded at 400 and several dendrites (>200 µm length) were randomly selected for each neuron in the MAP2 channel; on each dendrite, only clusters of >4 pixels were taken into consideration. Colocalization of IL-13 and IL-13Ra1 with synaptic markers was determined by tracing the contour of the IL-13/IL-13Ra1 clusters in the respective channels and thereafter verifying their overlap with the contour traced for the synaptic markers (VGLUT, Synaptophysin, Homer and PSD-95); a positive colocalization was assessed in case the area of the clusters overlapped >75%.

For the determination of active synapses in the synaptotagmin feeding assay as well as for the determination of synapses positive for surface NMDAR or AMPAR, confocal stacks of 7 0.5 µm-optical sections (acquired with a 63× NA 1.3 oil immersion objective, 1.5 optical zoom) were collapsed into maximum intensity projections; background was subtracted by rolling-ball method (20 pixels-radius). A more stringent threshold of 500/3500 (12-bit images) was applied; a cluster of >4 contiguous pixels which colocalized >75% with either only the presynaptic marker synaptophysin (for synaptotagmin) or both the pre- and postsynaptic marker Synaptophysin and PSD-95 (for surface NMDAR and AMPAR) was considered to define a “positive” synaptic site.

For the determination of the immunostaining intensity (Figs. 4, 5), confocal stacks of 10 0.5 µm-optical sections (acquired with a 40× NA 1.3 oil objective) were collapsed into maximum intensity projections; background was subtracted using the rolling-ball approach (50 pixels radius). A region of interest corresponding to the cell body was manually traced for each neuron using the MAP2 channel as template; the mean gray value was then extracted for the channel of the target under study (pCREB, pERK, DREAM, pIL-13Ra1, pSTAT6, pSTAT3, and ATF-3).

For the quantification of c-fos positive neurons, tile scan of single optical sections (acquired with a 20× NA 0.75 air objective) were merged and background-corrected as above; the c-fos channel was thresholded at 500, and neurons identified according to the MAP2, were considered fos+, if fos immunoreactivity occupied >50% of the nuclear area.

For the in situ hybridization experiment, confocal stacks of 9 1 µm-optical sections (acquired with a 40× NA 0.75 oil immersion objective) were collapsed in maximum intensity projections; background was subtracted by rolling-ball method (10 pixels diameter). The contour of each neuron (region of interest, ROI) was manually traced using the DAPI and VGLUT1 or 2 channels; for each ROI the mean gray value was obtained. For a more quantitative analysis, the number of mRNA spots was determined: the ROI corresponding to each neuron were extracted and analyzed using a custom-made ImageJ macro by applying the TrackMate plugin (ImageJ analysis tool^72^) with following parameters: radius: 0.4 pixel and threshold: 300, for single mRNA counting, after which the density was calculated as mRNA spots per 100 μm2.

For the quantification of the STED images, analysis was performed with the ImageJ software; the intensity plot was traced along a reference line starting from the axis of the dendrite and crossing the midline of the pre- or postsynaptic reference marker cluster; the distance from the axis of the dendrite to the peak of the target proteins was computed; in a separate analysis, the peak of the pre- or postsynaptic marker was considered as reference, and the peak of the target protein (IL-13, IL-13Ra1) was used as a landmark to measure the distance from the synaptic marker.

### Plasmids, AAV vectors and chemogenetics

Plasmids and AAV vectors have been previously described^32,37,73^. Details are reported in Supplementary Methods.

### Intracerebral injection of viruses

Intracortical injection of AAVs was performed, as previously reported^66^, in mice aged P30–P35. Details are reported in the Supplementary Methods file.

### Holotomography live imaging

The holotomographic microscope (Nanolive)^40^ was used to obtain high-contrast, label-free, time-lapsed imaging of cultured cortical neurons for 12 h, acquiring a three-dimensional holotomographic stack every 15 min, acquired with a 60× (NA 0.8) air objective. During the imaging, neuronal cultures (grown on optical-grade 35 mm dishes; Ibidi, Munich) were kept at 37 °C in a 95/5% O_2_/CO_2_ environment. Neuronal cultures were exposed to vehicles or, to simulate an acute excitotoxic environment associated with TBI, to 20 µM or 40 µM glutamate (added to the culture no more than 5 min before the beginning of the time-lapse imaging) either alone or in presence of IL-13 (50 ng/ml and 450 ng/ml; added at the same time of glutamate addition). We treated the cells with additional STAT6 inhibitor AS1517499 (1 µM, Axon Medchem) and JAK inhibitor Ruxolitinib (280 nM, Cayman) 30 min before the glutamate treatment. Images were exported using the STEVE software suite (Nanolive) and a custom macro was used to export the single timeframes into a single TIFF and video file. Nuclear pyknosis was calculated by thresholding the nucleus of every death cell (last frame) and assessing the frame (timepoint) when 50% of the nucleus was filled by this threshold.

### Human patients’ cohort

The recruitment of patients and the use of brain and CSF samples were authorized by the Kaifeng Central Hospital and the Alfred Hospital Human Ethics Committee respectively with approval No. 194-05 and the analysis at Ulm University was authorized by the Ulm University Ethical committee. Normal human cortex samples were obtained in agreement with the procedures approved by the Ulm University ethical committee with approval No. 245/17, informed and written consent was obtained from all body donors (Full details available in Supplementary Tables 1, 2, and 3 and in Supplementary Methods).

### Single-molecule array determination of cytokines in CSF

Single-molecule arrays were performed as per manufacturer instructions (Quanterix) for the quantification of TNF-α and IL-13. CSF samples were diluted 1:5 for TNF-α measurements and undiluted for IL-13 measurements.

### Darrow red Pigment-Nissl and Immunohistochemistry of human cortex and imaging

Darrow red Pigment-Nissl stain and Immunohistochemistry was performed on 50 µm thick cortical sections as described before^74^. Detailed protocol is reported in Supplementary Methods.

### Statistical analysis

Data analysis was performed using Graphpad prism version 8 software. Statistical analysis was performed using animals/cultures as a biological unit; whenever appropriate, single datapoints and averages per animal/culture are depicted in the graphs. All datasets were tested for normality using the Shapiro–Wilks test. Grouped analysis was performed using ANOVA with Dunnett’s or Sidak’s multiple correction or Kruskal–Wallis test with Dunn’s multiple correction, depending on normality. Kaplan–Meier curves were analyzed using the Log-rank (Mantel.Cox) test. When appropriate an unpaired *t*-test or Mann–Whitney test was used for comparing two groups. The timeline in the human ‘Melbourne cohort’ was analyzed using the Friedman test with Dunn’s multiple correction. Data were shown as scatterplot and mean or mean ± SD (WB data), both the average per experimental run (N) as well as single neurons or dendrites (n) were shown, and statistical significance was set at *p* < 0.05.

### Reporting summary

Further information on research design is available in the Nature Portfolio Reporting Summary linked to this article.

## Supplementary information

Supplementary Information

Reporting Summary

## Data Availability

All data presented in this study are available in a source data file, which are provided with this paper. The phosphor-proteomics data generated in this study have been deposited in the Figshare database under accession code 10.6084/m9.figshare.21758552. Source data are provided with this paper.

## Source data

Source Data

## Code availability

For protein array analyses, the code has been made publicly available on open-access GitHub repository PROTEAS (PROTein array Expression AnalysiS; https://github.com/Rida-Rehman/PROTEAS). The code for the analysis of the phospho-proteomics data is available under accession code 10.5281/zenodo.7470193.
