# Supplementary material for: Interleukin-13 and its receptor are synaptic proteins involved in plasticity and neuroprotection
Source: Nat Commun. 2023 Jan 13;14:200. doi: 10.1038/s41467-023-35806-8 (PMC9839781; doi:10.1038/s41467-023-35806-8)
Supplement: Supplementary file 1 — Supplementary Information [file 41467_2023_35806_MOESM1_ESM.pdf]

Supplementary Information

Interleukin-13 and its receptor are synaptic proteins involved in plasticity and neuroprotection

Shun Li, Florian olde Heuvel, Rida Rehman, Oumayma Aousji, Albrecht Froehlich, Zhenghui Li, Rebecca Jark, Wanhong Zhang, Alison Conquest, Sarah Woelfle, Michael Schoen, Caitlin C O´Meara, Richard Lee Reinhardt, David Voehringer, Jan Kassubek, Albert Ludolph, Markus Huber-Lang, Bernd Knöll, Maria Cristina Morganti-Kossmann, Marisa M. Brockmann, Tobias Boeckers, Francesco Roselli

Content:

Supplementary Methods

Supplementary Tables S1-S5

Supplementary Figures S1-S10

Supplementary methods

1. *Single-molecule mRNA in situ hybridization*

Brain sections were affixed on glass slides for histology. Target retrieval was performed by boiling the sections for 5 min in target retrieval solution (provided by ACDbio). Brain sections were washed twice with ddH_2O and once with ethanol. After which, the slides were pretreated with protease reagent III at 40°C for 30 min and probe hybridization for IL-13, VGLUT1, VGLUT2 and VGAT was performed at 40°C for 4.5 h. The slides were washed twice with washing buffer for 2 min and then incubated with amplification-1 reagent at 40°C for 30 min. After two washes for 2 min each, slides were incubated with amplification-2 reagent at 40°C for 15min, followed by two washes for 2 min each. The last amplification was performed with a 30 min incubation with amplification-3 followed by 2 washes for 2 min each. The final detection amplification was performed by incubating the amplification-4 reagent at 40°C for 45 min, and the final washing step was extended to 10 min each time. The co-immunostaining procedure followed immediately after this. The sections were blocked with PBS-/- containing 3% BSA and 0.3% Triton-X 100 at RT for 1 h and then incubated with primary antibody (Supplementary Table 1) at 4°C overnight. After being washed with PBS-/- 3x30 min, sections were incubated with secondary antibody (Supplementary Table S4) at RT for 2 h, followed by the last round of washing. Finally, the sections were mounted with Fluorogold prolong antifade mounting medium (Invitrogen).

2. *Quantitative PCR*

Culture dishes were washed three times with ice cold DPBS-/-. 0.5 ml QIAzol Lysis Reagent (Qiagen) was added per well in the 6-well plate. The pipette was used to rinse the bottom of dishes until the cell lysate was uniformly homogeneous. The tube containing the homogenate was placed at RT for 5 min. 0.15 ml chloroform was added and the tube was shaken vigorously for 15 sec. The tube containing the homogenate was kept at RT for 10 min. After centrifugating at 4°C, 12,000 x g for 15 min, the upper, aqueous phase was transferred into a new tube. The RNA was precipitated by vortexing with 250ul isopropanol. After 10 min at RT, the tube was centrifuged at 4°C, 12,000 x g for 10 min. The supernatant was discarded. After adding 1 ml of 75% ethanol, the tube was centrifuge at 4°C, 8000 x g for 10 min. The supernatant was removed completely, and the RNA pellet was air-dried. The RNA pellet was dissolved in 15-25 µl of RNase-free water. The concentration and quality were detected by NanoDrop 1000 (Thermo Scientific). The ratio of A260/A280 should be greater than 1.8. Reverse transcription was performed as described previously[8]. Briefly, 1 µg RNA was diluted with RNase free water to a volume of 40 µl, in addition 5 µl of randon hexamer were added, and incubated for 10 min at 70°C. 15 µl master mix was added, containing 12 µl RT 5x buffer, 2 µl dNTPs, 0.5 µl RiboLock Rnase Inhibitor (Thermo Scientific) and 0.5 µl M-MLV RT RNase (Promega). After incubating for 10 min at RT, the samples were incubated in the water bath at 42°C for 45 min. To stop the reaction, the samples were heated at 99°C for 5 min and placed on ice. The cDNA was used immediately for qPCR or store at -20 °C. 2 µl cDNA was mixed with 5 µl TB Green Premix Ex Taq (Takara Biosciences) and 3 µl primer mix (Supplementary Table S5) in a 96-well plate and measured with a Light Cycler 480II (Roche). All the samples were duplicated 6 times. We used the housekeeping gene GAPDH as a control. The CT values obtained from the light cycler were calculated with the $2^{-\Delta\Delta Ct}$ formula.

3. Primary rat cortical neurons culture

Each pregnant rat (Sprague-Dawley rats, obtained from Janvier Laboratories) had 6-15 embryos, and both sexes were used. After anesthesia with CO_2, adult pregnant rats were sacrificed. Laparotomy was performed to extract the embryos (E17-E18). The heads of embryos were removed into a sterile dish with ice cold Hank's Balanced Salt Solution (HBSS). After removing the skull, the frontal cortical tissues were manually dissected in ice cold HBSS under a stereomicroscope. The cortical tissues were incubated with 0.25% Trypsin/EDTA (1x) at 37°C and 5% CO_2 for 7-15 min. After three washes with Dulbecco's Modified Eagle Medium-high glucose (4.5 g/L) (DMEM) with 10% fetal bovine serum (FBS), 1% Glutamine (100x) and 1% Penicillin/Streptomycin (P/S) (DMEM+++), the brain tissues were mechanically dissociated in DMEM+++. The cells were resuspended in DMEM+++ after being filtered by a 100 μm sieve. The cortical cells were plated on ibidi dishes (μ-Dish 35 mm) (for Holotomography Live imaging), 6-wells plate (for cell harvest, WB and qPCR) or coverslips in the 24-wells plate (for Immunofluorescence) with DMEM+++. The dishes, plates and coverslips were coated with Poly-L-lysine (0.05 mg/ml) overnight at 37°C and 5% CO_2. DMEM+++ was replaced with Neurobasal medium supplemented with 2% B-27, 1% Glutamine and 1% P/S (NB+++) after 4-6 h. Cortical cells were grown in a humidified atmosphere at 37°C containing 5% CO_2 and medium was half-renewed weekly.

4. Immunocytochemistry of cultured rat cortical neurons

After treatment, the coverslips were washed two times with ice cold DPBS-/- and fixed for 15 min with ice cold 4% PFA added with 0.1 M sucrose. The cells were washed 2 times with ice cold DPBS-/- and blocked in PBS-/- with 3% BSA and 0.3% Triton-X 100 at RT for 2 h. Subsequently, cells were incubated with primary antibodies (Supplementary Table 4) diluted in blocking buffer at 4°C for 48 h. After washing with PBS-/- three times for 30 min, cells were incubated with secondary antibodies (Supplementary Table 4) at RT for 2 h and washed three times for 30 min with PBS-/- at RT. Finally, the coverslips were mounted on microscope glass slides with Fluorogold prolong antifade mounting medium (Invitrogen).

5. Intracerebral AAV injection

Mice were administered buprenorphine (0.05 mg/kg; Reckitt Beckshire Healthcare, Beckshire, UK) and meloxicam (1.0 mg/kg; Böhringer Ingelheim, Biberach an der Riß, Germany) 20 min before the surgery. Mice were positioned into a stereotactic frame under continuous isoflurane anesthesia (4% isoflurane in 96% O_2). After incising the scalp at the midline, a burr hole was drilled at the coordinates $x = +2.0$, $y = -2.0$ by a hand micro-drill, which corresponded to the somatosensory cortex. Approximately 200–500 nL of the viral suspension which was mixed with an equal volume of 1.5% Fast green solution, was injected by a pulled glass capillary which connected to a Picospritzer microfluidic device, over a span of 10 min. In case of preventing the backflow of the virus, the capillary was kept in place for another 10 min. To allow the bone to heal, the burr hole was left open. Prolene 7.0 surgical thread was used to suture the skin. Animals were transferred to a recovery cage with a warmed surface and ad libitum access to food and water after surgery. During the following 72 h, animals were administered additional doses of buprenorphine. The animals were monitored for possible neurological adverse events.

6. Phospho-Antibody Array assay

Neuroscience Phospho Antibody Array was performed as described in the manufacturer's instructions (Full Moon BioSystems). After three times washing with ice-cold DPBS-/-, 50 µl/6-well lysis buffer (RIPA + Protease inhibitor 3x + PhosSTOP 3x) was added and the cells were scraped on ice. The samples were treated with sonication on ice (5 sec, 5 x 10% cycle, 65% power). The samples were kept on ice for 30 min. After being centrifuged at 4°C at 12,000 x g for 20 min, the supernatant was gently transferred into a new tube. The concentration of protein was detected by BCA Protein Assay Kit (Thermofischer; Germany). 100 µg of protein was used for this array. The volume of the sample was brought to 75 µl by Labeling Buffer. After adding 3 µl of the Biotin/DMF solution, the mixture was incubated at RT for 2 h with vortexing every 10 min. 35 µl of Stop Reagent was added to stop labeling. The Antibody Arrays were blocked with blocking solution for 45 min on the shaker rotating at 55 rpm at RT. After blocking, the slide was washed with Milli-Q grade water ten times. The biotinylated sample was combined with 6 ml Coupling Solution. The slide was incubated with protein coupling mix on the orbital shaker rotating at 35 rpm for 2 h at RT. After coupling, the slide was washed with 1x Washing Solution on the shaker rotating at 55 rpm for 3x 10 min. The slide was extensively rinsed with Milli-Q water as before. The slide was submerged in the Detection Buffer with 0.5 mg/ml Cy5-streptavidin on the shaker rotating at 35 rpm for 20 min at RT in the dark. The slide was washed by Washing Solution and rinsed with Milli-Q water as before. After drying with compressed nitrogen, the slide was scanned using a GenePix 4000B array scanner (Molecular Devices, LLC).

7. *Brain fractionation and Western blot*

After dissection, the cortical tissue was suspended in Buffer 1 (10 mM HEPES pH 7.4, 2 mM EDTA, 5 mM Sodium orthovandate, 30 mM Sodium fluoride, 20 mM β-glycerolphosphate, protease inhibitor cocktail (Roche)) and homogenized with the Teflon douncer. 75 µl sample (Ho) was taken into a new tube called Ho. To remove extracellular matrix, nuclei and cell debris, the obtained homogenate (Ho) was centrifuged at 4°C, 500 x g for 5 min. The supernatant (S1) was collected in a new tube called P3. A 75 µl sample (S1) was taken into a new tube called S1. P3 was centrifuged at 4°C, 10,000 x g for 15 min to separate the crude membrane fraction (P2) from the cytosol (S2), which was collected. The pellet (P2) was resuspended in 500 µl Buffer 2 (10 mM HEPES pH 7.4, 2 mM EDTA, 2 mM EGTA, 5 mM Sodium orthovandate, 30 mM Sodium fluoride, 20 mM β-glycerolphosphate, 1% TritonX, protease inhibitor cocktail (Roche)). P2 was centrifuged at 4°C, 20,000 x g for 80 min. The supernatant (S3) contained the soluble synaptic cytosol. The pellet (P3) containing the insoluble PSD fraction was resuspended in Buffer 3 (50 mM Tris pH 9, 5 mM Sodium orthovandate, 30 mM Sodium fluoride, 20 mM β-glycerolphosphate, 1% NaDOC, protease inhibitor cocktail (Roche)).

BCA Protein Assay Kit (Thermo Fisher) was used to determine the protein concentrations as described in the manufacturer's instructions. 40-50 µg protein with 1 x loading buffer and 5% 2-Mercaptoethanol was heated at 95°C for 5 min. Samples were loaded on 8-12% SDS-PAGE gel and transferred to the nitrocellulose membrane. The membranes were blocked and then incubated with primary antibodies (Supplementary Table S4) on the shaker at 4°C overnight. After washing 5 times for 5 min with TBST on the shaker at RT, the membranes were incubated in horseradish secondary antibodies (Supplementary Table S4) in blocking buffer on the shaker at RT. The membranes were washed 5 times for 5 min again. Proteins bound to western blotting membranes were detected by Enhanced chemiluminescence (ECL). ImageJ was used to acquire the mean gray value of the bands. Mean gray value was measured for each band and enrichment fraction

was calculated to compute the percentage of IL-13 and IL-13Ra1 in both the P3 and S3 fraction, shown by PSD95 and Synaptophysin respectively. Alternatively, mean gray value was computed and normalised to loading control to depict differences in treatment or knock-out.

8. *Electrophysiological recordings.*

Whole-cell patch clamp recordings were obtained on excitatory hippocampal autaptic neurons at RT at day of differentiation (DIV) 15-20 at RT using a Multiclamp 700B amplifier (Axon Instruments) controlled by Clampex 9.2 software (Molecular Devices). Membrane capacitance and series resistance were compensated by 70% data filtered by low-pass Bessel filter at 3 kHz and sampled at 10 kHz using an Axon Digidata 1322A digitizer (Molecular Devices). Only recordings with a series resistance smaller than 10 MΩ were analyzed offline using AxoGraph (Axograph Instruments).

IL-13 treatment (50 ng/ml) was given into the culture media 30 min before the recording. During the recording, neurons were perfused continuously with the extracellular solution by a fast perfusion system (SF-77B; Warner Instruments) that contained the following (in mM): 140 NaCl, 2.4 KCl, 10 HEPES (Merck, NJ, USA), 10 glucose (Carl Roth, Karlsruhe, Germany), 2 $CaCl_2$ (Sigma-Aldrich, St. Louis, USA), and 4 $MgCl_2$ (Carl Roth) (~300mOsm; pH7.4). Borosilicate glass pipettes with a tip resistance of 2-3.5 MΩ and filled with the following internal solution (in mM): 136 KCl, 17.8 HEPES, 1 EGTA, 4.6 $MgCl_2$, 4 Na_2ATP, 0.3 Na_2GTP, 12 creatine phosphate, and 50 U/ml phosphocreatine kinase (~300 mOsm; pH7.4). Neurons were clamped at -70mV and excitatory postsynaptic currents (EPSC) were triggered by a 2 ms depolarization to 0mV. Spontaneous events (mEPSCs) were detected for 60 s at -70 mV.

9. *Plasmids, AAV Vectors and Chemogenetics*

The membrane-trafficked HaloTag (HaloTag-TM) as previously described[31] (kind gift from Michael Tadross). The pAAV-FLEX-SaCas9-U6-sgGrin1 plasmid was a gift from Larry Zweifel (Addgene plasmid # 124852 ; http://n2t.net/addgene:124852 ; RRID:Addgene_124852).

AAV pmSyn1-EBFP-Cre was a gift from Hongkui Zeng (Addgene plasmid # 51507 ; http://n2t.net/addgene:51507 ; RRID:Addgene_51507). pAAV-hSyn-EGFP was a gift from Bryan Roth (Addgene viral prep # 50465-AAV2; http://n2t.net/addgene:50465 ; RRID:Addgene_50465). pAAV-hSyn-PV-nuclear localisation sequence (NLS)-mC was previously reported[74].

AAV9 expressing the inhibitory Pharmacologically Selective Activation Module (PSAM)[36,73] based on the pAAV-cba-flox-PSAM (Leu141Phe, Tyr116Phe) GlyR-WPRE (kind gift of Scott Sternson), was obtained from Vector Biolabs (Malvern-PA, US), at the titers of 6×10^{12} viral genomes/ml. The PSEM308 agonist, was obtained from Apex Scientific Inc. (Stony Brook-NY, USA) and dissolved in sterile saline, was administered by intraperitoneal injection 30 min before TBI, at the dose of 5 μg/g.

For the DART pharmacology experiment, transfection was performed at DIV 8 1 μg/ml of plasmid together with 10 μl/ml optifect (Invitrogen), at DIV 14 the cells were treated with YM90K-DART (100 nM; kind gift of Mike Tadross) for 2 h, washed out and followed by an IL-13 (50 ng/ml) treatment for 1 h and further processed as described above.

For the CRISPR experiment, at DIV 8, the cells were co-transfected with 0.5 μg/ml BFP-CRE expressing plasmid and 0.5 μg/ml CRISPR-Cas9-Grin1 plasmid according to the Optifect protocol (Invitrogen); at DIV 14 the cells were treated with IL-13 (50 ng/ml) for 1 h and further processed as described above.

10. *Darrow red Pigment-Nissl and Immunohistochemistry of human cortex and imaging*

Consecutive sections were stained with Darrow red Pigment Nissl stain, to identify neuronal cells, and Immunohistochemistry for IL-13 or IL-13Ra1. Darrow red Pigment-Nissl stain was performed by rinsing the tissue 5 times 5 min in ddH_2O, followed by a 2h incubation of Darrow red working solution, which consisted of 80% Darrow red stock solution (0.25 g/l Darrow red and 1.5% glacial acetic acid in ddH_2O) and 20% 0.2 mol sodium acetate. Sections were dehydrated 20 min in 96% ethanol and 2h in 100% isopropanol, followed by two short incubations (3-5 min) in Xylene. Sections were mounted with Histomount mounting medium (National Diagnostics). Immunohistochemistry staining was performed on 50 µm thick cortical sections as described before[75]. Briefly, sections underwent an antigen retrieval step in citrate buffer (pH=6) for 20 min at 100°C, followed by a blocking step in 5% BSA and 0,25% Triton-X 100 at RT for 90 min. After blocking, sections were incubated in primary antibody (Supplementary Table S4) overnight at RT diluted in Tris buffer. The following day, the sections were washed 3 times with Tris buffer and incubated at RT for 90 min with the corresponding secondary biotinylated antibody diluted in Tris buffer (Supplementary Table S4). The sections were then washed 3 times and treated with avidin-biotin complex solution (VECTASTAIN® Elite ABC-HRP Kit, Vector Lab, #PK-6100) for 90 min at RT. The color reaction was then conducted using 3,3-diaminobenzidine (DAB) solution. Sections were dehydrated gradually in ethanol series, cleared in Xylene twice, and finally mounted on microscope glass slides with Histomount mounting medium (National Diagnostics). All sections were imaged using a brightfield microscope (Keyence, BZ-X800), equipped with a 20x air and 100x oil objective, by making a tile scan image of the grey and white matter of the cortex. Darrow red Pigment-Nissl stained sections and Immunohistochemical stained sections were matched by recognition of anatomical landmarks.

11.Human patients' cohort

Patients of Kaifeng Cohort (Brain cohort) were recruited at the Kaifeng Central Hospital, China, between January 2018 and March 2022; informed consent was obtained from the next of the kin. Inclusion criteria were severe TBI with a post-resuscitation GCS ≤8 and the need for neurosurgery to remove hematoma or injured brain tissue. In the TBI cohort, brain samples were collected in the proximity of, but not part of the macroscopic necrotic or hemorrhagic lesion (as visually identified by the neurosurgeon) within 6.5 h after TBI and kept at -80°C. Exclusion criteria comprised pregnancy, neurodegenerative diseases, HIV and other chronic infection/inflammatory diseases, or history of TBI. Inclusion criteria for controls were non brain-injury or other insults such as cerebral hemorrhage, aneurysm or autoimmune, inflammatory, infectious or neurodegenerative diseases (Full details available in Supplementary Table S2). Patients for Melbourne Cohort were recruited at the Alfred Hospital, Melbourne; informed consent was obtained from the next of the kin. Inclusion criteria were severe TBI with a post-resuscitation GCS ≤8 (unless initial GCS >9 was followed by deterioration requiring intubation) and, upon CT imaging, the need for an extraventricular drain (EVD) for ICP monitoring and therapeutic drainage of CSF. CSF was collected over 24 h and kept at 4°C; samples were obtained on admission (day 0) and daily up to day 5 after injury. Within an hour from collection, samples were centrifuged at 2000g for 15 min at 4°C and stored at -80°C until analysis. Exclusion criteria comprised pregnancy, neurodegenerative diseases, HIV and other chronic infection/inflammatory diseases, or history of TBI. Out of the 42 TBI patients constituting the original cohort[40], we selected samples from 30 patients, depending

on the availability of three aliquots for day 0, day 1 and day 4 after injury (Full details available in Supplementary Table S3).

Supplementary Table S1: Demographics of the normal post-mortem human brain cohort (Ulm).

Normal human cohort (Ulm)	**Case information**
**N (M/F)**	3 (2/1)
**Age**	66, 71, 74

Supplementary Table S2: Clinico-demographic data of the patients in the Kaifeng cohort (cortical biopsies).

Kaifeng Cohort	**Control group**	**TBI group**
**N (M/F)**	34 (20/14)	29 (18/11)
**Age (years, median, range)**	54 (41-86)	60 (23-86)
**Glasgow Coma Scale (median, range)**	N/A	5 (3-8)
**Surgery time after TBI (h, median, range)**	N/A	4 (1-6.5)

Supplementary Table S3: Clinico-demographic data for the patients in the Melbourne cohort (CSF samples).

Melbourne cohort	**Control group***	**D0 group**	**longitudinal group**
**N (M/F)**	6 (2/4)	27 (19/8)	11 (7/4)
**Age (median, range)**	71 (53-85)	37 (23-55)	29 (23-42)
**Glasgow Coma Scale (median, range)**	N/A	6 (3-9)	6 (3-7)
**Injury Severity Score (median, range)**	N/A	35 (17-59)	43 (17-59)
**Glasgow Outcome Scale-Extended (median, range)**	N/A	3.5 (1-8)	3 (1-6)

Supplementary Table S4

Primary antibodies	Host	Company	Catalog nr.	Dilution (application
ATF-3	Rabbit	Sigma	HPA001562	1:1000 (IF)
β-actin (2D4H5)	Mouse	Proteintech	69009-I-Ig	1:10000 (WB)
Bassoon (SAP7F407)	Mouse	Enzo	SAP7F407	1:400 (IF)
c-fos (2H2)	Mouse	Abcam	ab208942	1:500 (IF)
CREB	Mouse	CST	86B10	1:1000 (WB)
CREB(phospho) (87G3)	Rabbit	CST	9198S	1:500 (IF), 1:1000 (WB)
ERK1/2(phospho)	Rabbit	CST	9101	1:200 (IF)
ERK1/2(phospho) (D13.14.4E)	Rabbit	CST	4370	1:200 (IF)
GFP	Chicken	Abcam	ab13970	1:1000 (IF), 1:500 (RNAscope)
GFAP (G-A-5)	Mouse	Sigma	G3893	1:500 (IF)
GluN1	Mouse	SySY	114011	1:1000 (IF)
GluR1 (extracellular)	Rabbit	Alomone Labs	AGC-004	1:200 (IF)
GluN1 (extracellular)	Rabbit	Alomone Labs	AGC-001	1:200 (IF)
Homer-1 b/c	Rabbit	SySy	160022	1:500 (IF)
Iba1	Rabbit	SySy	234003	1:1000 (IF)
IL-13 (A-9)	Mouse	Santa	SC-393365	1:50 (IF), 1:100 (WB), 1:200 (IHC)
IL-13	Rabbit	Bioss	bs-0560R	1:100 (IF)
IL-13Rα1	Rabbit	Invitrogen	PA5-50989	1:500 (IF, WB), 1:200 (IHC)
IL-13Rα1 (phospho)	Rabbit	Invitrogen	PA5-38607	1:250 (IF)
KCHiP3 (Dream)	Rabbit	Thermo	PA5-11665	1:25 (IF)
Map2	Chicken	Encor	CPCA-MAP2	1:1000 (IF)
Map2	Guinea pig	SySy	188004	1:1000 (IF)

Neu-N	Guinea pig	SySy	266004	1:500 (IF), 1:200 (RNAscope)
PSD-95 (6G6-1C9)	Mouse	Abcam	ab2723	1:500 (IF), 1:4000 (WB)
RFP (conjugated)	Camel	Nanotech	N0404-AT565-L	1:500 (IF)
Shank2	Rabbit	Homemade	SA5192	1:500 (IF)
STAT3(phospho) (3E2)	Mouse	CST	9138S	1:200 (IF)
STAT6(phospho)	Rabbit	Invitrogen	PA5-104892	1:250 (IF)
STAT6	Rabbit	CST	9362	1:1000 (WB)
Synaptotagmin	Rabbit	SySy	105 103C3	1:500 (IF)
Synaptophysin	Guinea pig	SySy	101004	1:500 (IF)
Synaptophysin	Rabbit	Abcam	ab14692	1:100 (IF), 1:20000 (WB)
β-Tubulin	Rabbit	Abcam	ab179513	1:10000 (WB)
VGAT	Guinea pig	SySy	131004	1:500 (IF)
VGLUT1	Guinea pig	SySy	135304	1:500 (IF)
VGLUT2	Guinea pig	SySy	135404	1:500 (IF)

**secondary antibodies**	**Host**	**Company**	**Catalog nr.**	**Dilution (application)**
Anti-chicken AF 405	Goat	Abcam	ab175674	1:500 (IF)
Anti-chicken AF 488	Donkey	Biotium	20166	1:500 (IF, RNAscope)
Anti-guinea pig AF 488	Goat	Invitrogen	A11073	1:500 (IF)
Anti-guinea pig CF 568	Donkey	Biotium	20377	1:500 (IF)
Anti-guinea pig AF 633	Donkey	Invitrogen	A21105	1:500 (RNAscope)
Anti-rabbit AF 488	Donkey	Invitrogen	A21206	1:500 (IF)
Anti-rabbit AF 568	Donkey	Invitrogen	A10042	1:500 (IF)
Anti-rabbit AF 647	Donkey	Invitrogen	A31573	1:500 (IF)

Anti-mouse AF 488	Donkey	Invitrogen	A21202	1:500 (IF)
Anti-mouse AF 647	Donkey	Invitrogen	A31571	1:500 (IF)
DAPI 405	-	Thermo	62247	1:1000 (IF)
FluoTag-X4 anti-Rabbit AberriorStar 580	-	Nanotag	N2404-Ab580-S	1:500 (IF, STED)
FluoTag-X2 anti-Mouse AberriorStar635	-	Nanotag	N1202-Ab635P-S	1:500 (IF; STED)
Anti-mouse	Horse	Vector Lab	PI-2000	1:3000 (WB), 1:200 (IHC)
Anti-rabbit	Goat	Vector Lab	PI-1000	1:5000 (WB), 1:200 (IHC)

Supplementary Table S5: List of genes and sequences used for RT-qPCR.	
**Gene (target)**	**Sequence**
c-fos (rat)	forward: 5’ -gagccgcgaacgagcagtga- 3’<br>Reverse: 5’ -ggcgaggggtccaggggtag- 3’
egr-1 (rat)	forward: 5’ -aacaaccctacgagcacctg- 3’<br>Reverse: 5’ -accagcgccttctcgttatt- 3’
egr-2 (rat)	forward: 5’ -aggccgtagacaaaatcccag- 3’<br>Reverse: 5’ -cctcccagttcaccattggg- 3’
fos-B (rat)	forward: 5’ -gtgagagatttgccagggtc- 3’<br>Reverse: 5’ -agagagaagccgtcaggttg- 3’
gadd45a (rat)	forward: 5’ -ggagtcagcgcaccataact- 3’<br>Reverse: 5’ -ggtcgtcatcttcatccgca- 3’
gadd45b (rat)	forward: 5’ -ctcctggtcacgaactgtcat- 3’<br>Reverse: 5’ -ggacccactggttattgcct- 3’
GAPDH (rat)	forward: 5’ -gacatgccgcctggagaaac- 3’<br>Reverse: 5’ -agcccaggatgccctttagt- 3’
IL-13 (rat)	forward: 5’ -atggtatggagcgtggacct- 3’<br>Reverse: 5’ -actggagatgttggtcaggg- 3’
IL-13Ra1 (rat)	forward: 5’ -tgagtctgctgtgaccgaac- 3’<br>Reverse: 5’ -atagttggtgtccgggcttg- 3’
IL-13Ra2 (rat)	forward: 5’ -cacagggccagactcaaagat- 3’<br>Reverse: 5’ -gtgggttcagggtcttccttt- 3’
BDNF (human)	forward: 5’ -catccgaggacaaggtggcttg- 3’<br>Reverse: 5’ -gccgaactttctggtcctcatc- 3’
GAPDH (human)	forward: 5’ -gtctcctctgacttcaacagcg- 3’<br>Reverse: 5’ -accaccctgttgctgtagccaa- 3’
IL-13 (human)	forward: 5’ -atgcatccgctcctcaatcc- 3’<br>Reverse: 5’ -agtgagagcaatgaccgtgg- 3’
IL-13Ra1 (human)	forward: 5’ -cctgaatgagaggatttgtctgc- 3’<br>Reverse: 5’ -cagtcacagcagactcaggatc- 3’
IL-13Ra2 (human)	forward: 5’ -gtggagtgataaacaatgctggg- 3’<br>Reverse: 5’ -tgggtaggtgtttggcttacgc- 3’

TNF-α (human)	forward: 5’ -ctcttctgcctgctgcactttg- 3’<br>Reverse: 5’ -atgggctacaggcttgtcactc- 3’

Supplementary Figure 1

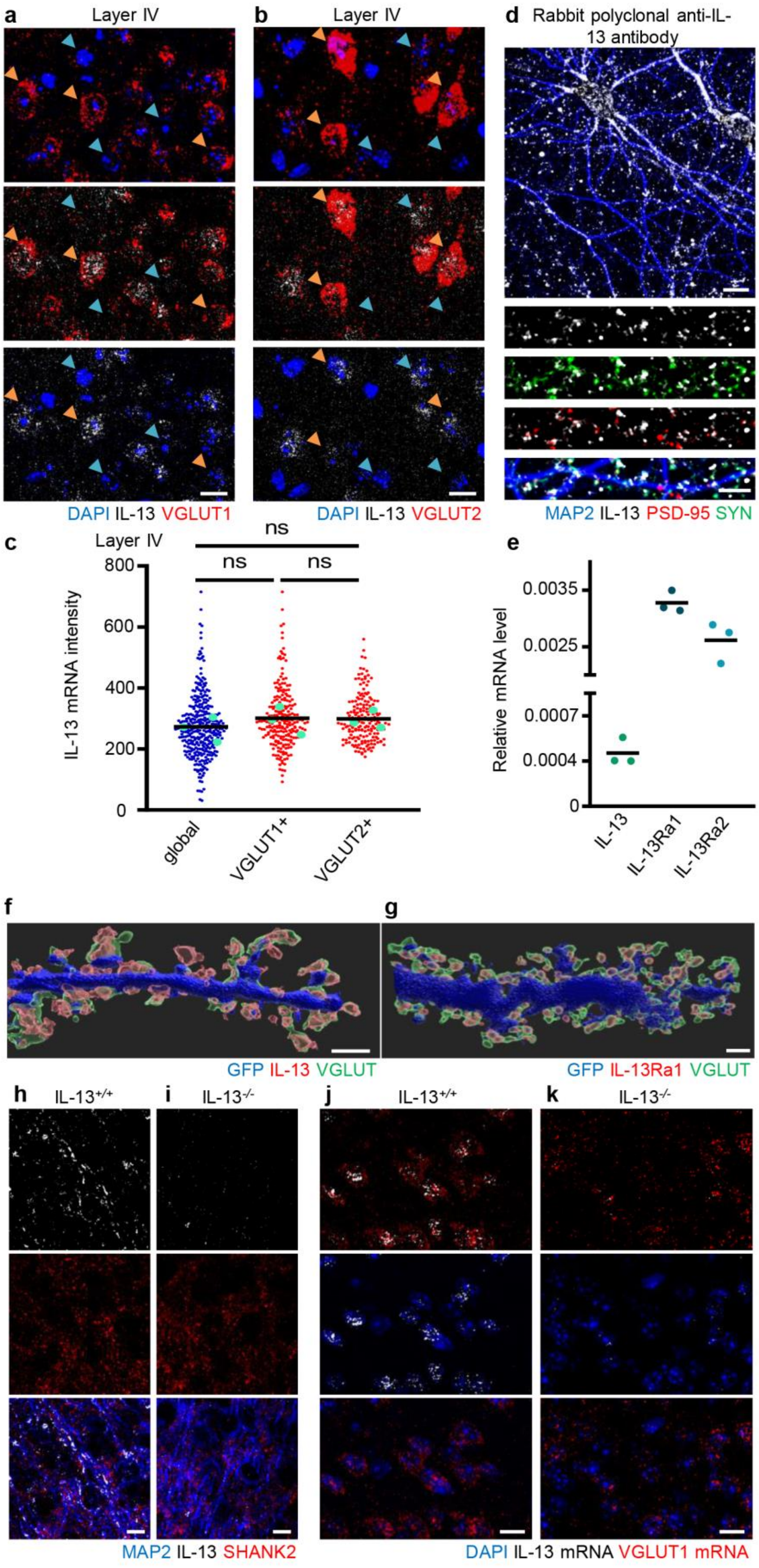

Supplementary Figure 1: Verification of neuronal IL-13 and IL-13Ra1 expression and synaptic localisation.

a-c. No significant increase in *IL-13* mRNA intensity in VGLUT1 positive glutamatergic neuronal population (orange arrows, panel a; p=0.8076), or VGLUT2 positive glutamatergic neuronal population (orange arrows, panel b; p=0.7944) compared to the global cell population (blue arrows) in layer IV of mouse cortical sections (single molecule in situ hybridisation). N = 3; n = global: 351; VGLUT1+: 220; VGLUT2+: 175 neurons. Scale bar: 20 μm.

d. Synaptic localisation of another IL-13 antibody in mouse cortical sections (Immunostaining with MAP2, pan-VGLUT and SHANK2). Scale bar overview 10 μm and insert 5 μm. N = 3.

e. Baseline mRNA levels of IL-13, IL-13Ra1 and IL-13Ra2 in rat primary cortical neurons (RT-qPCR). N = 3.

f-g. 3D Imaris reconstruction of a GFP labelled dendrite with pan-VGLUT and IL-13 or IL-13Ra1 staining shows high level of colocalization of IL-13 and IL-13Ra1 with presynaptic terminals on dendrites and spines. Scale bar: 2 μm.

h-i. IL-13 antibody validated on *IL-13*$^{-/-}$ and *IL-13*$^{+/+}$ littermates together with MAP2 and SHANK2 immunostaining. N = 3. Scale bar: 10 μm.

j-k. IL-13 ISH probe validated on *IL-13*$^{-/-}$ and *IL-13*$^{+/+}$ littermates together with VGLUT1 probe and counterstained with DAPI. N = 3. Scale bar: 20 μm.

(c): One-way ANOVA with Sidak’s multiple comparison. Source data are provided as a Source Data file.

Supplementary Figure 2

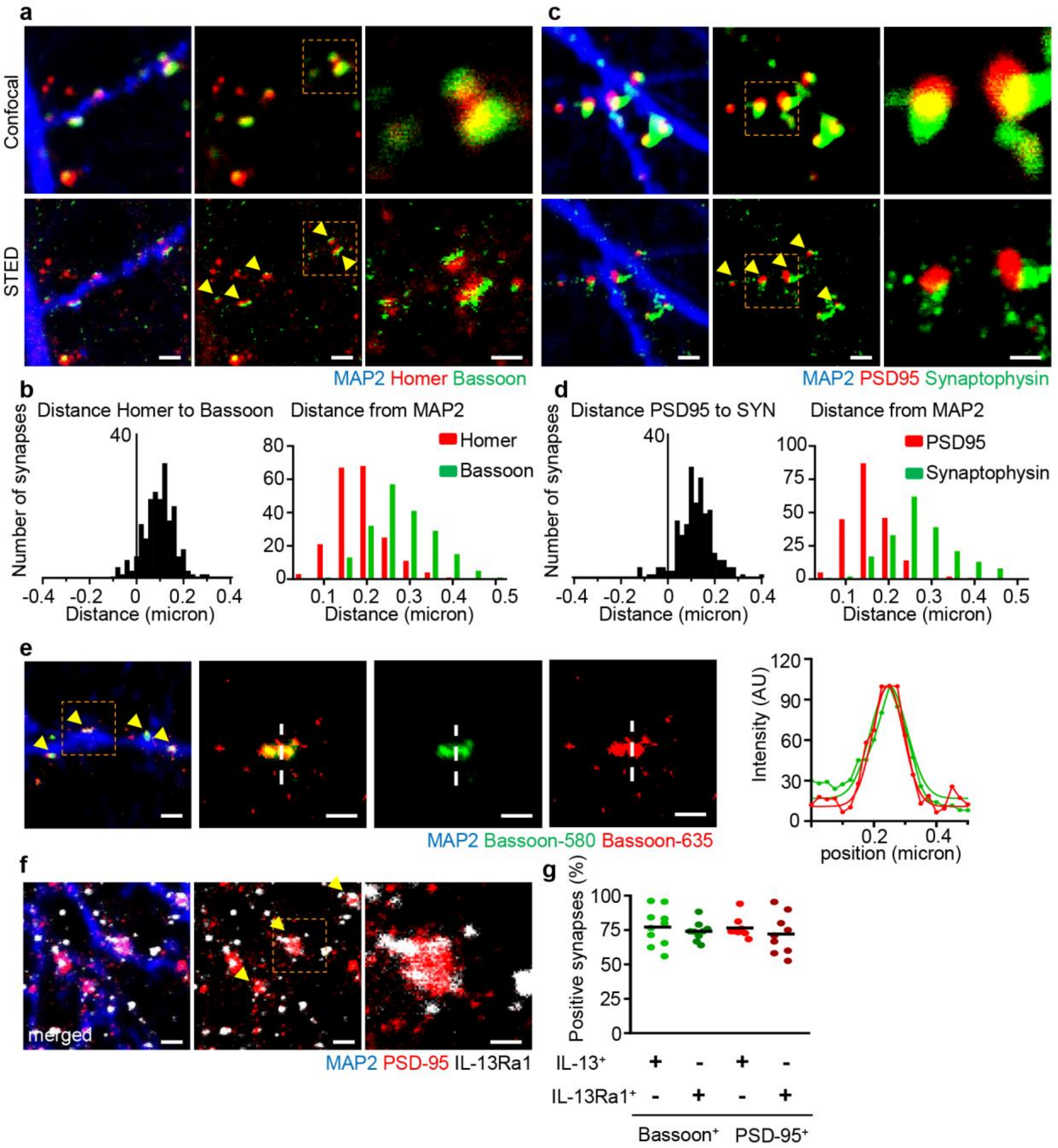

Supplementary Figure 2: Validation of pre- and postsynaptic localisation and STED imaging.

a. Validation of the resolution of the STED imaging: distinct separation of pre and postsynaptic markers Bassoon and Homer, barely visible in confocal imaging.

b. Distribution analysis confirms that Homer and Bassoon peaks do not overlap and are located, referencing the MAP2 profile of the dendrite, the former proximal and the latter distally, in agreement with their known post- and presynaptic location.

c. Validation of the resolution of the STED imaging: distinct separation of pre and postsynaptic markers Synaptophysin and Bassoon, barely visible in confocal imaging.

d. Distribution analysis confirms that PSD-95 and Synaptophysin peaks do not overlap and are located, referencing the MAP2 profile of the dendrite, the former proximal and the latter distally, in agreement with their known post- and presynaptic location.

e. Bassoon puncta immunostained with anti-Bassoon primary antibody and two anti-mouse secondary antibodies (conjugated with Abberior-Star580 and 635) display an almost complete overlap of the two channels, indicating no systematic shift in the imaging planes. AU: arbitrary units.

f. STED images of single synapses show multiple IL-13Ra1 puncta in a single PSD-95 spot.

g. IL-13 shows 77% and 76% synaptic colocalization with Bassoon and PSD-95 respectively. IL-13Ra1 shows 74% and 72% synaptic colocalization with Bassoon and PSD-95 respectively. N = 10 dendrites per colocalization.

In all experiments N = 3; n = 200 synapses. Scale bar overview: 1 µm, scale bar insert: 500 nm. Source data are provided as a Source Data file.

Supplementary Figure 3: List of proteins, phosphosites, Log FC and Adj. P-value for selected proteins from the array.

Complete list of significantly phosphorylated proteins can be found in supplementary information. (a, b): PROTein array Expression AnalysiS; github.com/Rida-Rehman/PROTEAS. Source data are provided as a Source Data file.

a

Protein	Phosphosite	Log FC	Adj. P-value
NMDAR1	S897	0.238	0.011
GluR1	S849	0.190	0.018
	S863	0.140	0.046
CaMK-II	T286	0.178	0.008
	T305	0.134	0.026
Trk B	Y515	0.084	0.026
	Y705	0.136	0.036
Synuclein alpha	Y133	0.125	0.031
	Y136	0.319	0.005
Synapsin1	S62	0.145	0.015
CaMK-I	S177	0.010	0.040
CaMK-IV	S196/200	0.093	0.018
GSK3 alpha/beta	Y216/279	0.200	0.017
PP2A alpha	Y307	0.220	0.027
PKA	T197	0.145	0.007
GRK2	S685	0.106	0.046
Tau	S231	0.144	0.018
Merlin	S518	0.136	0.029
Trk A	Y680	-0.113	0.018
	Y701	-0.115	0.013
	Y791	-0.507	0.006
Trk C	Y516	-0.323	0.039
GAB1	Y627	-0.211	0.007
GAB2	Y643	-0.364	0.015
NMDAR1	S896	-0.255	0.003

b

Protein	Phosphosite	Log FC	Adj. P-value
CaMK-II	T286	0.254	0.041
Synapsin1	S62	0.393	0.004
Synuclein alpha	Y133	0.284	0.018
	Y125	0.251	0.018
CaMK-I	T177	0.438	0.004
GAB1	Y659	0.236	0.016
DAB1	Y220	0.164	0.040
GRK2	S29	0.336	0.011
Tau	S235	0.272	0.029
	S356	0.261	0.046
	S422	0.137	0.044
Merlin	S10	0.192	0.035
GAP43	S41	0.129	0.047
NMDAR2A/2B	Y1246/1252	-0.161	0.018
NMDAR1	S895	-0.203	0.007
GluR4	S862	-0.207	0.009
MUNC-18a	S313	-0.211	0.029
Synapsin I	S305	-0.257	0.025
Parkin1	S131	-0.180	0.025
Ataxin1	S776	-0.176	0.028
Preselinin-1	S357	-0.270	0.031
Trk A	Y791	-0.354	0.004
Doublecortin	S297	-0.222	0.035

Supplementary Figure 4

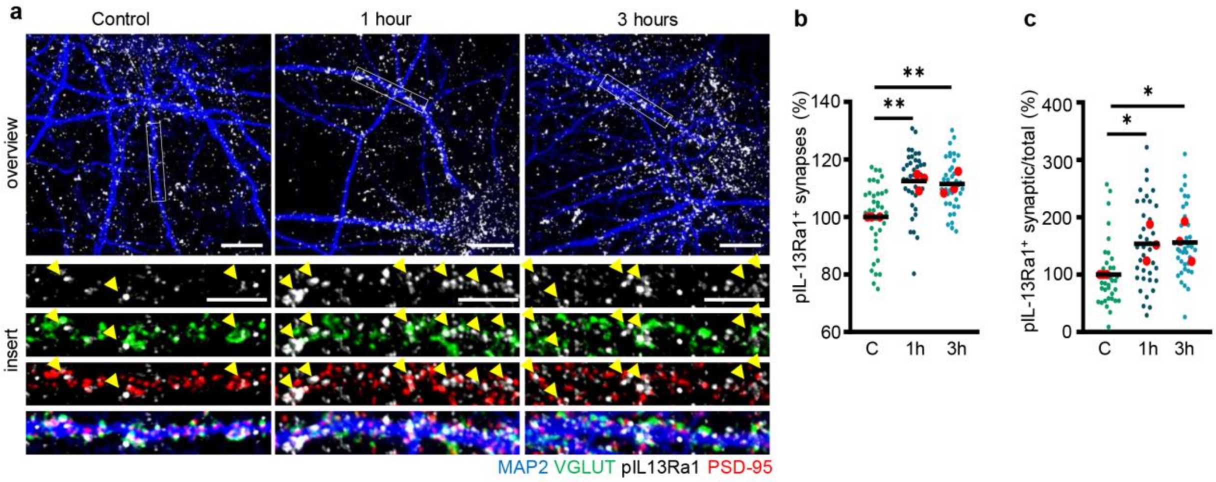

Supplementary Figure 4: Il-13 increases phosphorylation of IL-13Ra1 at mature synapses. a-c. Significant up-phosphorylation of IL-13Ra1 at mature synapses 3 h after IL-13 treatment (50 ng/ml); vehicle (0.1% BSA as control) (panel b: C vs 1 h: p=0.0033; C vs 3h: p=0.0052; panel c: C vs 1 h: p=0.0431; C vs 3h: p=0.0450). N = 3; n = 35-37 independent dendrites. Scale bar overview 10 µm and inset 5 µm. *: p<0.05. **: p<0.01.

(b, c): One-way ANOVA with Dunnet's multiple comparison. Source data are provided as a Source Data file.

Supplementary Figure 5

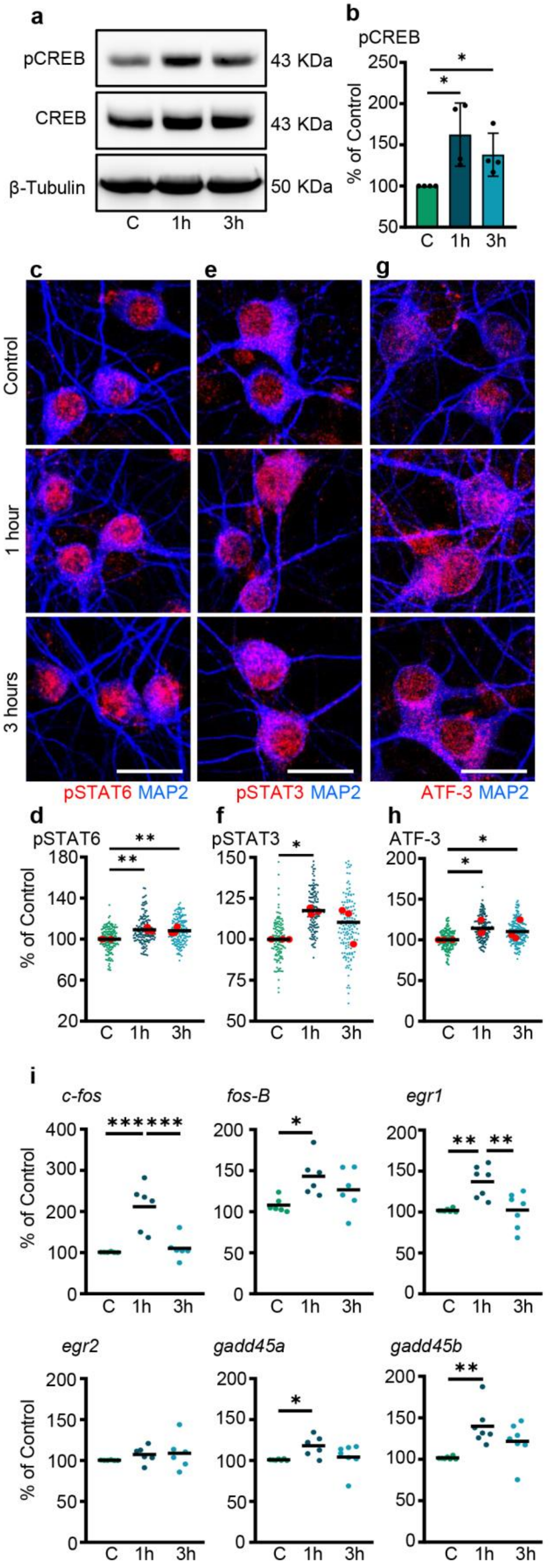

Supplementary Figure 5: IL-13 induces STAT phosphorylation and immediate-early genes transcription.

a-b. Significant up-phosphorylation of CREB in rat cortical neurons 1 h ($p=0.0173$) and 3 h ($p=0.0267$) after IL-13 treatment (50 ng/ml). N = 4. Data shown as mean±SD

c-d. Significant up-phosphorylation of STAT6 in rat cortical neurons 1 h ($p=0.0048$) and 3 h ($p=0.0087$) after IL-13 treatment (50 ng/ml). N = 3; n = C: 165; 1 h: 142; 3 h: 146 neurons.

e-f. Significant up-phosphorylation of STAT3 in rat cortical neurons 1 h ($p=0.0413$) after IL-13 treatment (50 ng/ml). N = 3; n = C: 128; 1 h: 137; 3 h: 150 neurons.

g-h. Significant up-phosphorylation of ATF-3 in rat cortical neurons 1 h ($p=0.0144$) and 3 h ($p=0.0270$) after IL-13 treatment (50 ng/ml). N = 3; n = C: 193; 1 h: 173; 3 h: 194 neurons.

i. Significant increase of *c-fos* ($p=0.0002$), *fos-B* ($p=0.0204$), *egr1* ($p=0.0019$), *gadd45a* ($p=0.0262$) and *gadd45b* ($p=0.0025$) expression 1 h after IL-13 treatment (50 ng/ml), with a subsequent decrease 3 h after treatment (RT-qPCR). N = 6.

Scale bar: 20 µm. In all experiments, vehicle (0.1% BSA) was used as control. *: $p<0.05$. **: $p<0.01$. ***: $p<0.001$.

(b): Two-tailed unpaired T-test. (d, f, h, i): One-way ANOVA with Dunnet’s multiple comparison. Source data are provided as a Source Data file.

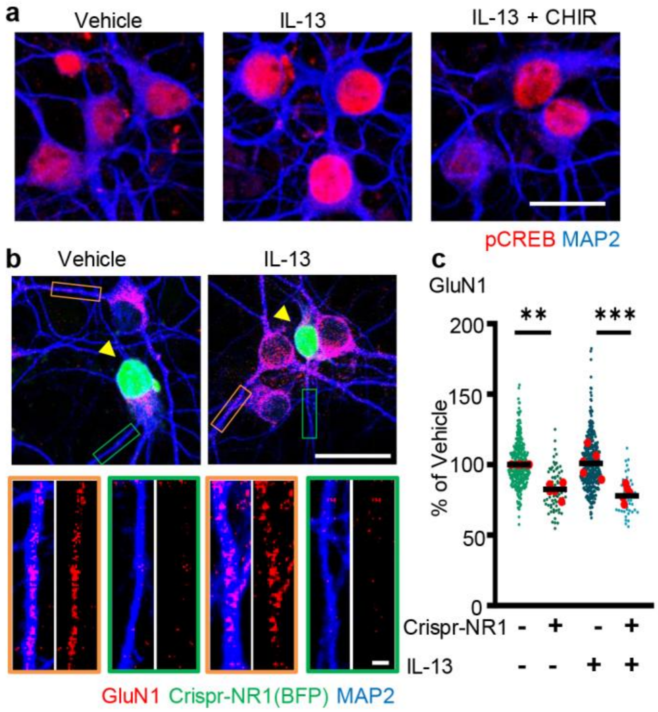

Supplementary Figure 6: Transfection of CRISPR-Cas9-Grin1 in neurons decreases GluN1 expression.

a. GSK-3 inhibitor (CHIR 98014; 10 µM) does not alter IL-13 induced CREB phosphorylation. 0.1% DMSO as control. Scale bar: 20 µm. N = 3; n = Veh: 282; IL-13: 301; CHIR: 251 neurons.

b-c. Grin1 knock down by CRISPR-Cas9 significantly decreases GluN1 expression in both vehicle and IL-13 treatment (yellow arrows; p=0.0012 and p=0.0002 respectively). 0.1% BSA as control. N = 5; n = BFP-/IL-13-: 324; BFP+/IL-13-: 77; BFP-/IL-13+: 366; BFP+/IL-13+: 46 neurons. Scale bar overview: 20 µm, scale bar insert: 2 µm. **: p<0.01; ***: p<0.001.

(c): One-Way ANOVA with Sidak's multiple comparison. Source data are provided as a Source Data file.

.

Supplementary Figure 7

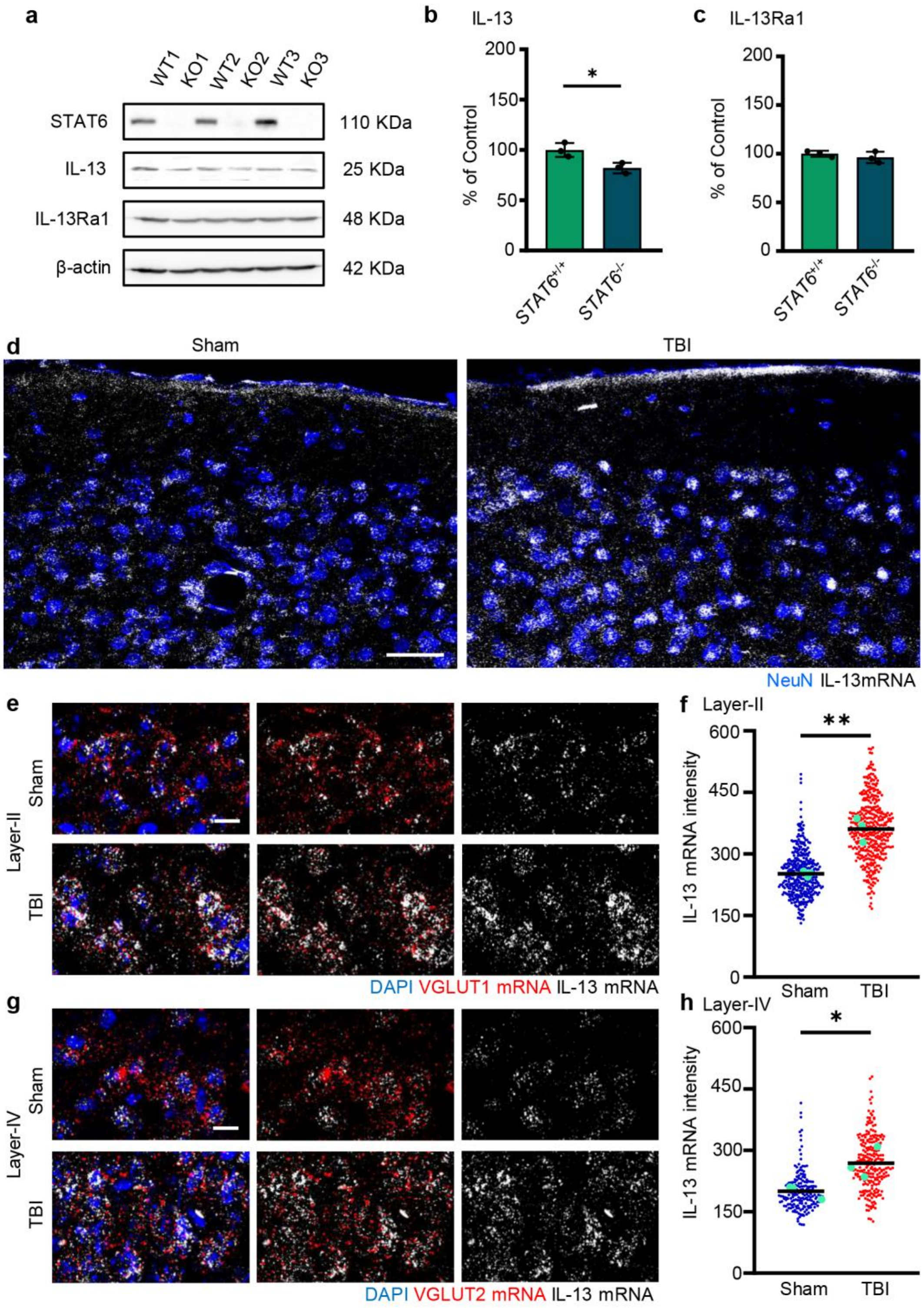

Supplementary Figure 7: IL-13 induction upon Trauma in distinct subpopulations of excitatory neurons.

a-c. Significant downregulation of IL-13 protein in *STAT6*$^{-/-}$ mice (p=0.0237), IL-13Ra1 is not affected in *STAT6*$^{-/-}$ mice (p=0.3723). N = 3. Data shown as mean±SD

d. Overview of the upregulation of *IL-13* mRNA expression in a blunt, closed traumatic brain injury murine model (3 h after injury). N = 3. Scale bar: 50 μm.

e-f. Significant increase of *IL-13* mRNA expression in VGLUT1 positive neurons after TBI (3h after injury; p=0.0035). N = 3; n = sham: 375; TBI: 401 neurons.

g-h. Significant increase of *IL-13* mRNA expression in VGLUT2 positive neurons after TBI (3 h after injury; p=0.0478). N = 3; n = sham: 179; TBI: 241 neurons. Scalebar 20 μm. *: p<0.05. **: p<0.01.

(b, c, f, h): Two-tailed unpaired T-test. Source data are provided as a Source Data file.

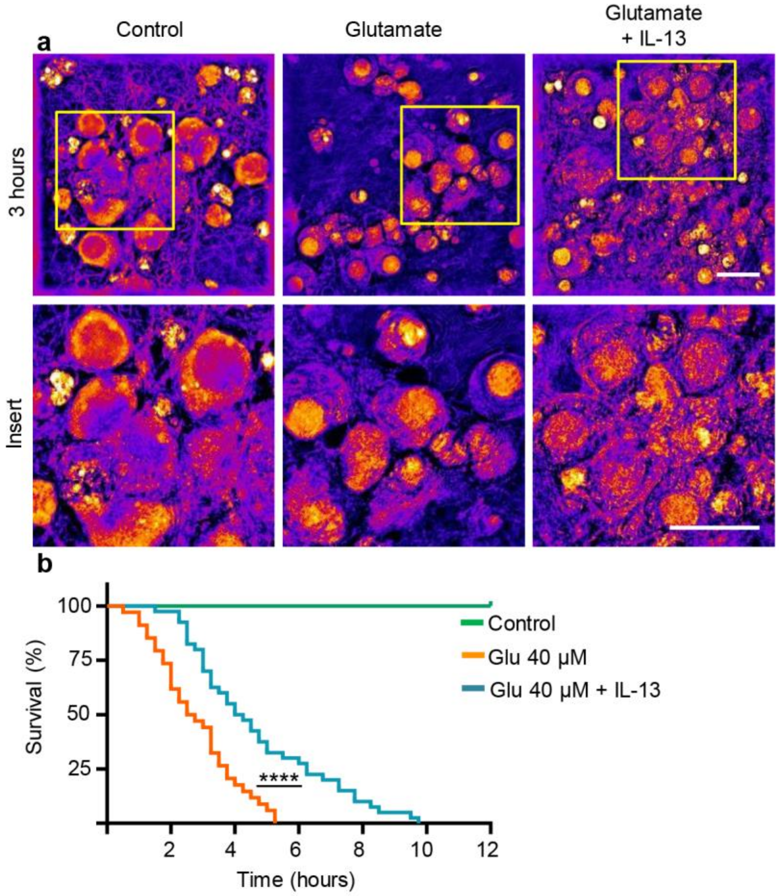

Supplementary Figure 8: IL-13 reduces high dose-glutamate excitotoxic neuronal death. a-b. Significant reduction of glutamate (40 μM) induced neuronal toxicity after IL-13 treatment (50 ng/ml) in rat cortical neurons (p<0.0001). N = 4; n =C: 40; 40 μM: 34; 40 μM+IL-13: 40 neurons. Scale bar: 20 μm. ****: p<0.0001.

(b): Log-rank (Mantel-Cox) test. Source data are provided as a Source Data file.

Supplementary Figure 9

Normal cortex samples

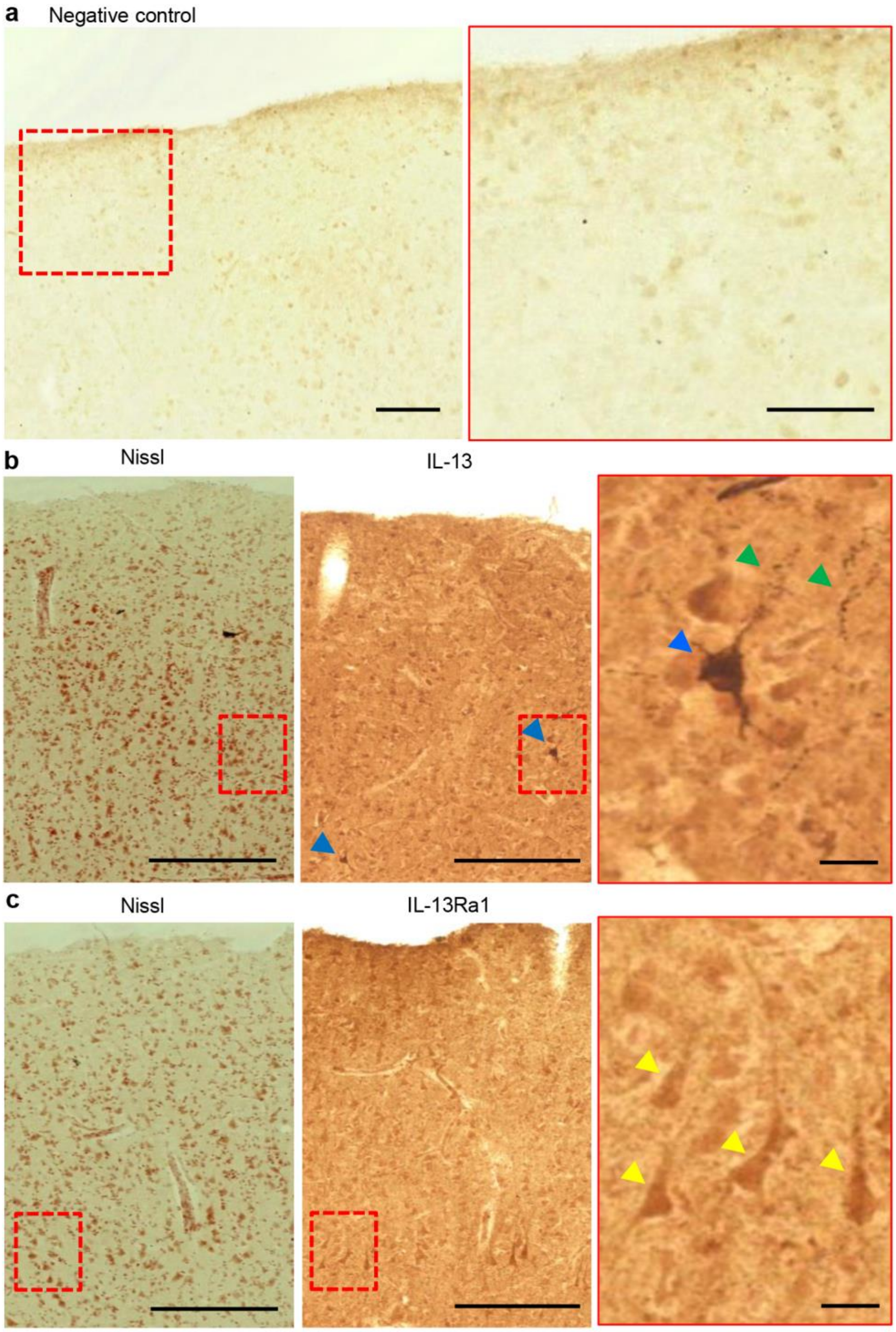

Supplementary Figure 9: Validation of antibodies on human cortex samples.

a. Negative-control for IL-13 immunohistochemical staining (no primary antibody, but secondary added) shows no signal in human post-mortem cortical tissue. N = 3. Scale bar overview: 100 µm, scale bar insert: 50 µm.

b. Nissl staining and IL-13 immunohistochemistry (left and middle panels, same as in figure 9); right panel: magnified view of a high-IL-13-expressing cell (blue arrowhead) with neuronal morphology; in the same field, punctate bead-string-like immunoreactivity for IL-13 (green arrowheads.

c. Nissl staining (left) and IL-13Ra1 immunohistochemistry (middle; same as in figure 9); right panel: magnified view of a group of cells with neuronal morphology, immunoreactive for IL-13Ra1 in cell body and dendrites.

b, c Scale bar overview: 100 µm, scale bar insert: 10 µm.

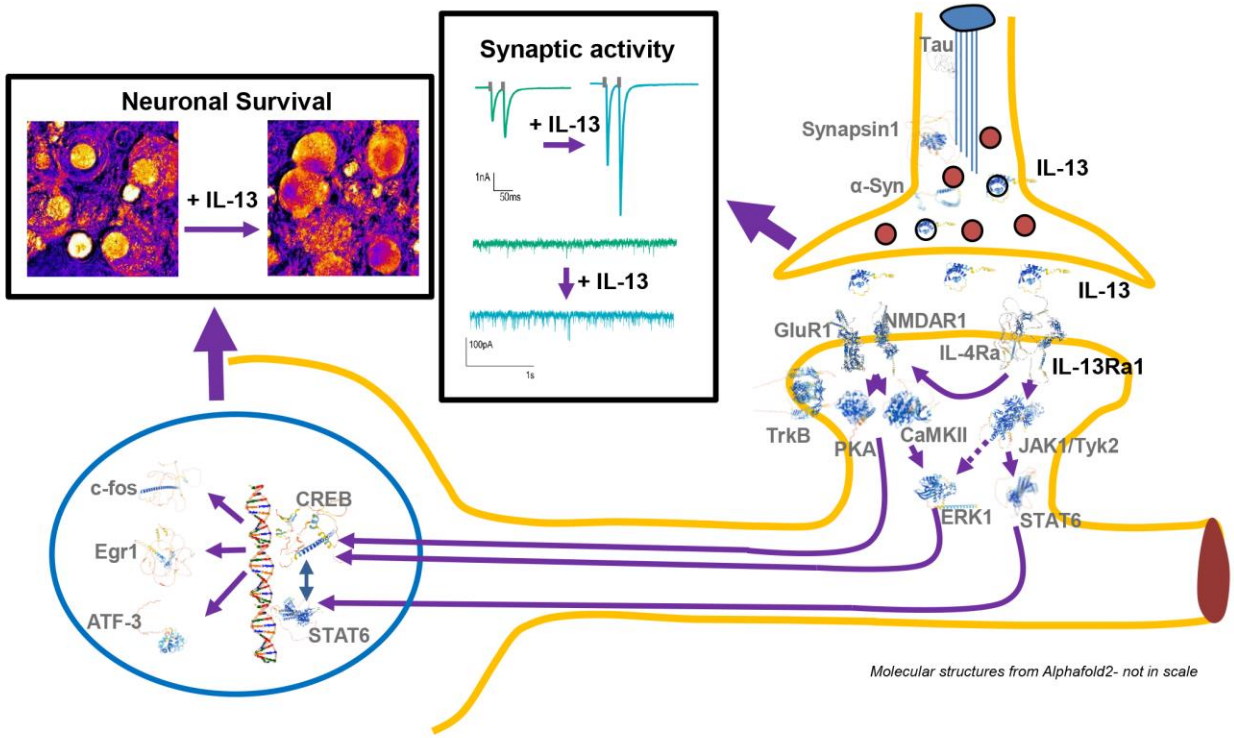

Supplementary Figure 10: IL-13/IL-13Ra1 are pre- and postsynaptic proteins, respectively, involved in the control of synaptic signalling and neuronal activity, which are reflected in transcriptional changes ultimately resulting in neuroprotective effects.
