# Supplementary material for: Interleukin-13 and its receptor are synaptic proteins involved in plasticity and neuroprotection
Source: Nat Commun. 2023 Jan 13;14:200. doi: 10.1038/s41467-023-35806-8 (PMC9839781; doi:10.1038/s41467-023-35806-8)
Supplement: Supplementary file 2 — Reporting Summary [file 41467_2023_35806_MOESM2_ESM.pdf]

# nature portfolio

Corresponding author(s): Prof. Dr. Francesco Roselli

Last updated by author(s): 20/12/22

# Reporting Summary

Nature Portfolio wishes to improve the reproducibility of the work that we publish. This form provides structure for consistency and transparency in reporting. For further information on Nature Portfolio policies, see our Editorial Policies and the Editorial Policy Checklist.

## Statistics

For all statistical analyses, confirm that the following items are present in the figure legend, table legend, main text, or Methods section.

| n/a | Confirmed | |
|---|---|---|
| ☐ | ☒ | The exact sample size ($n$) for each experimental group/condition, given as a discrete number and unit of measurement |
| ☐ | ☒ | A statement on whether measurements were taken from distinct samples or whether the same sample was measured repeatedly |
| ☐ | ☒ | The statistical test(s) used AND whether they are one- or two-sided<br>*Only common tests should be described solely by name; describe more complex techniques in the Methods section.* |
| ☒ | ☐ | A description of all covariates tested |
| ☐ | ☒ | A description of any assumptions or corrections, such as tests of normality and adjustment for multiple comparisons |
| ☐ | ☒ | A full description of the statistical parameters including central tendency (e.g. means) or other basic estimates (e.g. regression coefficient) AND variation (e.g. standard deviation) or associated estimates of uncertainty (e.g. confidence intervals) |
| ☐ | ☒ | For null hypothesis testing, the test statistic (e.g. $F$, $t$, $r$) with confidence intervals, effect sizes, degrees of freedom and $P$ value noted<br>*Give P values as exact values whenever suitable.* |
| ☒ | ☐ | For Bayesian analysis, information on the choice of priors and Markov chain Monte Carlo settings |
| ☒ | ☐ | For hierarchical and complex designs, identification of the appropriate level for tests and full reporting of outcomes |
| ☒ | ☐ | Estimates of effect sizes (e.g. Cohen's $d$, Pearson's $r$), indicating how they were calculated |

*Our web collection on statistics for biologists contains articles on many of the points above.*

## Software and code

Policy information about availability of computer code

| | |
|---|---|
| Data collection | Microscope Software Platform LAS X Life Science version 8; Zen Microscopy Software; GenePix Pro version 6.1; ImageLab version 6.0; Axograph; SIMOA HD-X analyser; Roche lightcycler 480II. |
| Data analysis | GraphPad Prism software version 8.0.1; ImageJ software version 1.53t; R software version 3.0; Imaris software version 9.9.0; Microsoft Office 365. For protein array analyses, the code has been made publicly available on open-access GitHub repository PROTEAS (PROTein array Expression AnalysiS; github.com/Rida-Rehman/PROTEAS) |

For manuscripts utilizing custom algorithms or software that are central to the research but not yet described in published literature, software must be made available to editors and reviewers. We strongly encourage code deposition in a community repository (e.g. GitHub). See the Nature Portfolio guidelines for submitting code & software for further information.

## Data

Policy information about availability of data

All manuscripts must include a data availability statement. This statement should provide the following information, where applicable:

- Accession codes, unique identifiers, or web links for publicly available datasets
- A description of any restrictions on data availability
- For clinical datasets or third party data, please ensure that the statement adheres to our policy

All raw data are provided wihtin a Source data file. Source data are provided with this paper.

# Field-specific reporting

Please select the one below that is the best fit for your research. If you are not sure, read the appropriate sections before making your selection.

☒ Life sciences ☐ Behavioural & social sciences ☐ Ecological, evolutionary & environmental sciences

For a reference copy of the document with all sections, see nature.com/documents/nr-reporting-summary-flat.pdf

# Life sciences study design

All studies must disclose on these points even when the disclosure is negative.

| | |
|---|---|
| Sample size | Due to the exploratory nature of this study, an a priori sample size determination was not possible. For the in vivo experiments, we anticipated an effect size of 2 (or larger), which for a total sample size of 12 would generate for a one-way ANOVA with 4 groups a minimum power of 94% for alpha=0.01. The sample size for the in vitro experiments adhere to the typical group sizes in the field and were chosen based on experiences with these samples and the chosen read-outs. |
| Data exclusions | For the in vitro and in vivo data no data was excluded from the analysis. For the human data, two data point were excluded from the control group after identifying outliers using the 'Rout' method in the Graphpad prism software suite. |
| Replication | All experiments were replicated in 3-6 independent cultures or in 3-4 independent animals per group. whenever appropriate, the average per culture or per animal is depicted along the raw data for each neuron/dendrite taken into consideration. |
| Randomization | All samples and mice were randomized by numbering the samples and using a automated randomization tool in microsoft excel. |
| Blinding | Analysis was performed by numbering all samples, mice or pictures by one experimentator and letting the second experimentator analyse the samples, mice or pictures without being aware of the treatment. Groups and numbers were allocated after analysis was finished. |

# Reporting for specific materials, systems and methods

We require information from authors about some types of materials, experimental systems and methods used in many studies. Here, indicate whether each material, system or method listed is relevant to your study. If you are not sure if a list item applies to your research, read the appropriate section before selecting a response.

## Materials & experimental systems

| n/a | Involved in the study | |
|---|---|---|
| ☐ | ☒ | Antibodies |
| ☒ | ☐ | Eukaryotic cell lines |
| ☒ | ☐ | Palaeontology and archaeology |
| ☐ | ☒ | Animals and other organisms |
| ☐ | ☒ | Human research participants |
| ☒ | ☐ | Clinical data |
| ☒ | ☐ | Dual use research of concern |

## Methods

| n/a | Involved in the study | |
|---|---|---|
| ☒ | ☐ | ChIP-seq |
| ☒ | ☐ | Flow cytometry |
| ☒ | ☐ | MRI-based neuroimaging |

## Antibodies

Antibodies used

Primary antibodies Host Company Catalog nr. Dilution (application
ATF-3 Rabbit Sigma HPA001562 1:1000 (IF)
β-actin (2D4H5) Mouse Proteintech 69009-I-Ig 1:10000 (WB)
Bassoon (SAP7F407) Mouse Enzo SAP7F407 1:400 (IF)
c-fos (2H2) Mouse Abcam ab208942 1:500 (IF)
CREB Mouse CST 86B10 1:1000 (WB)
CREB(phospho) (87G3) Rabbit CST 9198S 1:500 (IF), 1:1000 (WB)
ERK1/2(phospho) Rabbit CST 9101 1:200 (IF)
ERK1/2(phospho) (D13.14.4E) Rabbit CST 4370 1:200 (IF)
GFP Chicken Abcam ab13970 1:1000 (IF), 1:500 (RNAscope)
GluN1 Mouse SySY 114011 1:1000 (IF)
GluR1 (extracellular) Rabbit Alomone Labs AGC-004 1:200 (IF)
GluN1 (extracellular) Rabbit Alomone Labs AGC-001 1:200 (IF)
Homer-1 b/c Rabbit SySy 160022 1:500 (IF)
IL-13 (A-9) Mouse Santa SC-393365 1:50 (IF), 1:100 (WB), 1:200 (IHC)
IL-13 Rabbit Bioss bs-0560R 1:100 (IF)
IL-13Rα1 Rabbit Invitrogen PA5-50989 1:500 (IF, WB), 1:200 (IHC)
IL-13Rα1 (phospho) Rabbit Invitrogen PA5-38607 1:250 (IF)

KCHiP3 (Dream) Rabbit Thermo PA5-11665 1:25 (IF)
Map2 Chicken Encor CPCA-MAP2 1:1000 (IF)
Map2 Guinea pig SySy 188004 1:1000 (IF)
Neu-N Guinea pig SySy 266004 1:500 (IF), 1:200 (RNAscope)
PSD-95 (6G6-1C9) Mouse Abcam ab2723 1:500 (IF), 1:4000 (WB)
RFP (conjugated) Camel Nanotech N0404-AT565-L 1:500 (IF)
Shank2 Rabbit Homemade SA5192 1:500 (IF)
STAT3(phospho) (3E2) Mouse CST 9138S 1:200 (IF)
STAT6(phospho) Rabbit Invitrogen PA5-104892 1:250 (IF)
STAT6 Rabbit CST 9362 1:1000 (WB)
Synaptotagmin Rabbit SySy 105 103C3 1:500 (IF)
Synaptophysin Guinea pig SySy 101004 1:500 (IF)
Synaptophysin Rabbit Abcam ab14692 1:100 (IF), 1:20000 (WB)
β-Tubulin Rabbit Abcam ab179513 1:10000 (WB)
VGAT Guinea pig SySy 131004 1:500 (IF)
VGLUT1 Guinea pig SySy 135304 1:500 (IF)
VGLUT2 Guinea pig SySy 135404 1:500 (IF)

secondary antibodies Host Company Catalog nr. Dilution (application)
Anti-chicken AF 405 Goat Abcam ab175674 1:500 (IF)
Anti-chicken AF 488 Donkey Biotium 20166 1:500 (IF, RNAscope)
Anti-guinea pig AF 488 Goat Invitrogen A11073 1:500 (IF)
Anti-guinea pig CF 568 Donkey Biotium 20377 1:500 (IF)
Anti-guinea pig AF 633 Donkey Invitrogen A21105 1:500 (RNAscope)
Anti-rabbit AF 488 Donkey Invitrogen A21206 1:500 (IF)
Anti-rabbit AF 568 Donkey Invitrogen A10042 1:500 (IF)
Anti-rabbit AF 647 Donkey Invitrogen A31573 1:500 (IF)
Anti-mouse AF 488 Donkey Invitrogen A21202 1:500 (IF)
Anti-mouse AF 647 Donkey Invitrogen A31571 1:500 (IF)
DAPI 405 - Thermo 62247 1:1000 (IF)
FluoTag-X4 anti-Rabbit AberriorStar 580 - Nanotag N2404-Ab580-S 1:500 (IF, STED)
FluoTag-X2 anti-Mouse AberriorStar635 - Nanotag N1202-Ab635P-S 1:500 (IF; STED)
Anti-mouse Horse Vector Lab PI-2000 1:3000 (WB), 1:200 (IHC)
Anti-rabbit Goat Vector Lab PI-1000 1:5000 (WB), 1:200 (IHC)

The complete list of all antibodies used has additionally been added to the manuscript as a supplementary table

Validation

Validation of the two IL-13 antibodies have been performed on IL-13-KO mouse samples (shown in supplementary Figure 1h-i) and by using only the secondary antibody without adding the primary. The homemade shank2 antibody has been validated in KO animals and published previously (PMID: 22699619). The pCREB antibody has been validated using SimpleChIP® Enzymatic Chromatin IP Kits, provided by the company (CST).
All other antibodies were validated by the companies for their use in respective applications and by in dept comparison with known and published results or available literature. Validation methods used by companies vary depending on the company and antibody produced.

The following companies provide statements regarding validation procedures of their antibodies:
- Abcam (https://www.abcam.com/primary-antibodies/how-we-validate-our-antibodies#IHC%20and%20ICC),
- Invitrogen (https://www.qa4.thermofisher.com/de/de/home/life-science/antibodies/invitrogen-antibody-validation.html),
- Alomone Labs (https://www.alomone.com/why-alomone-labs), SySy (https://www.sysy.com/resources/antibody-validation),
- Nano-tag (https://nano-tag.com/),
- Cell Signaling Technologies (https://www.cellsignal.de/about-us/cst-antibody-validation-principles).

Furthermore, we have extensive experience with the antibodies used in this study, they have been subject to previous publications (PMID: 31207335; PMID: 32484501; PMID: 29982364; PMID: 29774782; PMID: 36403069).

## Animals and other organisms

Policy information about studies involving animals; ARRIVE guidelines recommended for reporting animal research

Laboratory animals

Species: Mus Musculus; strain: C57BL/6J, all genetic mouse lines(IL-13-KO, STAT6-KO and PV-cre) used in this study were bred on a B6 background strain; sex: male; ages: p60
Genetic mouse lines used:
- WT mice: B6SJLF1/J
- STAT6-KO: B6.129S2(C)-Stat6tm1Gru/J
- IL-13-KO: Il13tm1.1Anjm
- PV-cre: B6.129P2-Pvalbtm1(cre)Arbr/J

Wild animals

The study did not involve wild animals

Field-collected samples

The study did not involve samples collected from the field

Ethics oversight

All animal experiments were approved by the Ulm University Veterinary and the animal experimentation oversight comittee at the Regierungspräsidium Tübingen under Licence Nr.: O.103-12 and 1420. The animal experiments on IL-13-KO mice were approved by the American Association for Laboratory Animal Science (IACUC) and the local animal committee under licence Nr.: AUA00005516.

Note that full information on the approval of the study protocol must also be provided in the manuscript.

## Human research participants

Policy information about studies involving human research participants

| | |
|---|---|
| Population characteristics | Supplementary Table 1. Full details normal human cohort (Ulm)<br>Normal human cohort (Ulm) Case information<br>N (M/F): 3 (2/1)<br>Age: 66, 71, 74<br><br>Supplementary Table 2. Patient information Kaifeng cohort<br>Kaifeng Cohort: Control group - TBI group<br>N (M/F): 34(20/14) - 29 (18/11)<br>Age (median, range): 54 (41-86) - 60 (23-86)<br>Glasgow Coma Scale (median, range): N/A - 5 (3-8)<br>Surgery time after TBI (h): N/A - 4 (1-6.5)<br><br>Supplementary Table 3. Patient information Melbourne cohort.<br>Melbourne cohort: Control group* - D0 group - longitudinal group<br>N (M/F): 6 (2/4) - 27 (19/8) - 11 (7/4)<br>Age (median, range): 71 (53-85) - 37 (23-55) - 29 (23-42)<br>Glasgow Coma Scale (median, range): N/A - 6 (3-9) - 6 (3-7)<br>Injury Severity Score (median, range): N/A - 35 (17-59) - 43 (17-59)<br>Glasgow Outcome Scale-Extended (median, range): N/A - 3.5 (1-8) - 3 (1-6)<br><br>Full patient information have been added to the manuscript as supplementary tables. |
| Recruitment | Patients of Kaifeng Cohort were recruited at the Kaifeng Central Hospital, China, between January 2018 and January 2021; informed consent was obtained from the next of the kin. Inclusion criteria were severe TBI with a post-resuscitation GCS ≤8 and the need for neurosurgery to remove hematoma or injured brain tissue. In the TBI cohort, brain samples were collected in the proximity of, but not part of the macroscopic necrotic or hemorrhagic lesion (as visually identified by the neurosurgeon) within 6.5 h after TBI and kept at -80°C. Exclusion criteria comprised pregnancy, neurodegenerative diseases, HIV and other chronic infection/inflammatory diseases, or history of TBI. Inclusion criteria for controls were: non brain-injury or other insults such as cerebral hemorrhage, aneurysm or autoimmune, inflammatory, infectious or neurodegenerative diseases<br><br>Patients for Melbourne Cohort were recruited at the Alfred Hospital, Melbourne; informed consent was obtained from the next of the kin. Inclusion criteria were: severe TBI with a post-resuscitation GCS ≤8 (unless initial GCS>9 was followed by deterioration requiring intubation) and, upon CT imaging, the need for an extraventricular drain (EVD) for ICP monitoring and therapeutic drainage of CSF. CSF was collected over 24h and kept at 4°C; samples were obtained on admission (day 0) and daily up to day 5 after injury. Within an hour from collection, samples were centrifuged at 2000g for 15 min at 4°C and stored at -80°C until analysis. Exclusion criteria comprised pregnancy, neurodegenerative diseases, HIV and other chronic infection/ inflammatory diseases, or history of TBI. Out of the 42 TBI patients constituting the original cohort |
| Ethics oversight | Normal human cortex samples were obtained in agreement with the procedures approved by the Ulm University ethical committee with approval No. 245/17, informed and written consent was obtained from all body donors.<br><br>The recruitment of patients and the use of brain and CSF samples were authorized by the Kaifeng Central Hospital and Alfred Hospital Human Ethics Committee with approval No. 2019LL001-KFZXYY and 194-05 respectively and the analysis at Ulm University was authorized by the Ulm University Ethical committee.<br><br>The ethical approval documents are available and can be provided upon request. |

Note that full information on the approval of the study protocol must also be provided in the manuscript.
